# Current Status and Perspectives of Dual-Atom Catalysts Towards Sustainable Energy Utilization

**DOI:** 10.1007/s40820-024-01347-y

**Published:** 2024-02-29

**Authors:** Yizhe Li, Yajie Li, Hao Sun, Liyao Gao, Xiangrong Jin, Yaping Li, Zhi LV, Lijun Xu, Wen Liu, Xiaoming Sun

**Affiliations:** 1 State Key Laboratory of Chemical Resource Engineering, Beijing Advanced Innovation Center for Soft Matter Science Engineering, College of Chemistry, Beijing University of Chemical Technology, Beijing, 100029 People’s Republic of China; 2 Xinjiang Coal Mine Mechanical and Electrical Engineering Technology Research Center, Xinjiang Institute of Engineering, Ürümqi, 830023 Xinjiang Uygur Autonomous Region People’s Republic of China

**Keywords:** Dual-atom catalysts, Synergetic effect, Electrocatalysis, Oxygen reduction reaction, CO_2_ reduction reaction, Hydrogen evolution reaction, N_2_ reduction reaction

## Abstract

The exploration of sustainable energy utilization requires the implementation of advanced electrochemical devices for efficient energy conversion and storage, which are enabled by the usage of cost-effective, high-performance electrocatalysts. Currently, heterogeneous atomically dispersed catalysts are considered as potential candidates for a wide range of applications. Compared to conventional catalysts, atomically dispersed metal atoms in carbon-based catalysts have more unsaturated coordination sites, quantum size effect, and strong metal–support interactions, resulting in exceptional catalytic activity. Of these, dual-atomic catalysts (DACs) have attracted extensive attention due to the additional synergistic effect between two adjacent metal atoms. DACs have the advantages of full active site exposure, high selectivity, theoretical 100% atom utilization, and the ability to break the scaling relationship of adsorption free energy on active sites. In this review, we summarize recent research advancement of DACs, which includes (1) the comprehensive understanding of the synergy between atomic pairs; (2) the synthesis of DACs; (3) characterization methods, especially aberration-corrected scanning transmission electron microscopy and synchrotron spectroscopy; and (4) electrochemical energy-related applications. The last part focuses on great potential for the electrochemical catalysis of energy-related small molecules, such as oxygen reduction reaction, CO_2_ reduction reaction, hydrogen evolution reaction, and N_2_ reduction reaction. The future research challenges and opportunities are also raised in prospective section.

## Highlights

- The advancement and current status of dual-atom catalysts are reported.
- The synergistic effects exhibited by recent dual-atom catalysts in mechanistic studies are classified and summarized.
- Challenges and prospects of dual-atom catalysts in synthesis, characterization, applications, and theory are discussed.

## Graphical abstract

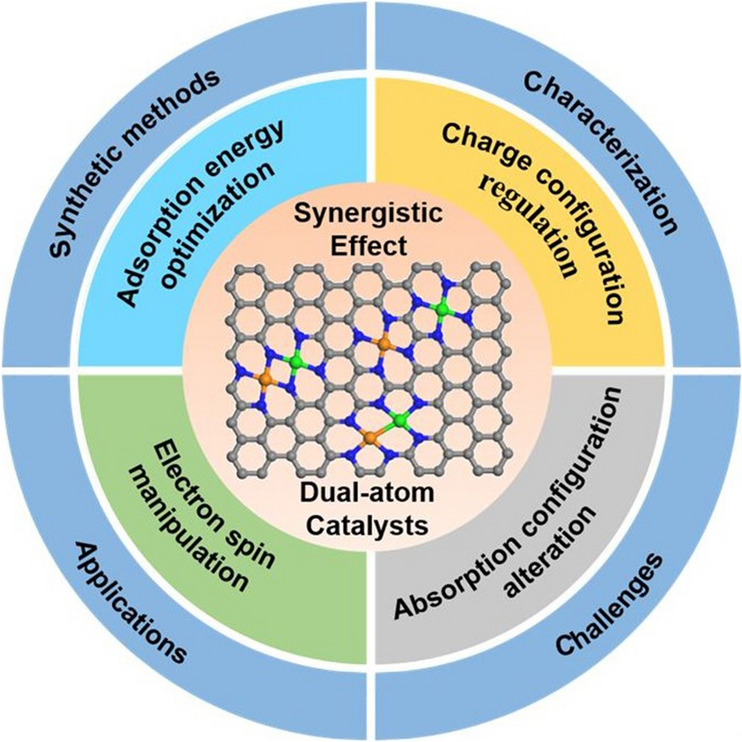

## Introduction

Energy and environmental problems are becoming more and more prominent, along with the rapid progress of urbanization and industrialization in human society. One of the important causes is the overutilization of fossil fuels such as oil, coal, and natural gas. It is imperative to develop sustainable and clean energy technologies, so that energy shortage and environmental degradation can be mitigated [1–4]. The electrochemical conversion and utilization of energy-related small molecules has received great attention over the past two decades. The usage of clean electricity to convert earth-abundant and available molecules such as O_2_, CO_2_, N_2_, H_2_O into fuels or value-added products could go a long way towards reducing the consumption of fossil fuels and ultimately achieving a carbon neutral society [5–7]. However, the electrochemical reaction process for these small molecules requires a large overpotential, which results in the loss of energy efficiency [8–10]. Therefore, the development of inexpensive and efficient electrocatalysts has become one of the key research areas in the forefront of electrochemistry and materials chemistry.

In previous research, electrocatalysts suffer from the problems of insufficient activity, high cost, poor selectivity, poor stability, and susceptibility to poisoning [11–14]. In addition, the catalytic mechanisms of electrocatalysts are not clear due to the limitations of preparation method and environmental factors. On this basis, a variety of new electrocatalysts have been proposed and applied to practical applications. In these efforts, the atomically dispersed electrocatalysts, with their theoretical maximum atomic utilization and highly tunable electronic properties, have attracted great interest around the world [15–17]. Among them, single-atomic catalysts (SACs) supported on carbon skeleton have become one of the hottest research areas (the atomically dispersed catalysts discussed in this paper are all based on carbon skeleton). SACs are substantially cost-effective due to the extraordinarily theoretical high utilization of metal atoms and hence offer a broad application prospective in the industrial domain. SACs have made a large number of breakthroughs in a variety of electrocatalytic reactions in recent years, especially in the reduction reactions of small molecules such as oxygen reduction reaction (ORR) [18], CO_2_ reduction reaction (CO_2_RR) [19], hydrogen evolution reaction (HER) [20], and N_2_ reduction reaction (NRR) [21]. In order to satisfy the application needs in different fields, researchers have performed a series of modifications on nitrogen-doped carbon-loaded single-atom catalysts [22], such as coordination engineering [23], defect engineering [24], geometric modulation [25], and long-range synergy [26, 27]. Among them, the construction of another single-atom site in the neighbourhood of a single-atom site to form dual-atom catalysts (DACs) is considered as a promising regulatory mechanism that can provide new opportunities for the application of SACs [28, 29].

**Fig. 1:**
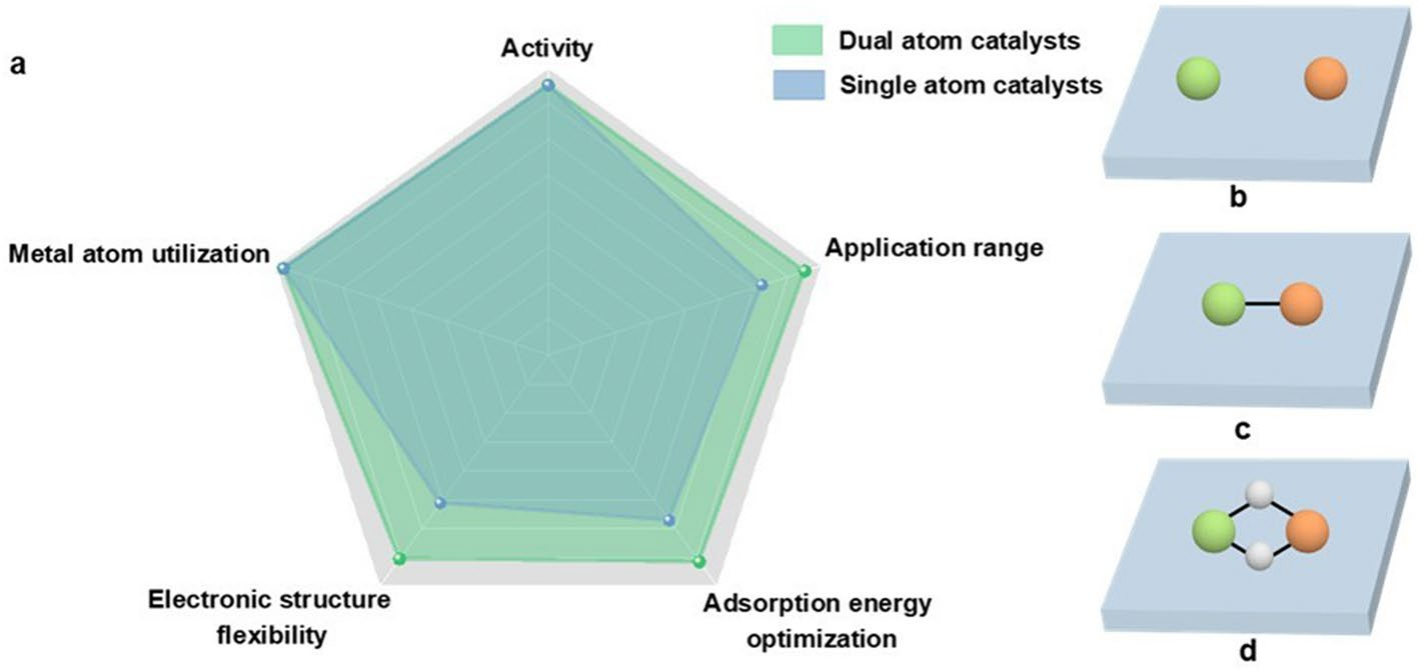
**a** Advantages of dual-atom catalysts over single-atom catalysts. **b–d** Schematic diagram of the classification of DACs. The green ball and the orange ball represent the metal atoms M and M’, respectively. The white ball represents N or O atom. The blue cube represents the substrate

DACs, like SACs, have a theoretical 100% metal atom utilization and high activity. In addition, the combination of pairs based on a rich library of metal atoms to form diatomic sites can greatly increase the structural diversity of catalysts, thus providing convenience in catalyst design and screening (Fig. 1a). The neighbouring metal atoms could make effort in tuning electronic structure. Wang et al. developed atomic dispersed electrocatalysts of Fe–N–C and Fe_2_–N–C. Through constructing Fe_2_–N–C, a negative d band centre position was obtained. A lower energy gap between antibonding and bonding states in *CO adsorption was achieved by the orbital coupling [30]. The Fe_2_–N–C could maintain high Faradaic efficiency and better durability over a wide potential range. The two metal atoms constituting the active site in DACs are promising to simultaneously adsorb reactant molecules to serve reactions requiring dual-site catalysis, thus broadening application range of SACs. The adjacent two metal sites could lead to different reaction pathways to intervene the selectivity. In electroreduction of carbon dioxide or carbon monoxide, a C_2_ pathway with C–C coupling procedure always requires contiguous metal active sites. Li et al. developed dual-atomic Cu sites which was benefit for the coupling of two absorbed CO molecules [31]. As a result, the Cu–Cu sites could efficiently electrocatalytic CO reduction to C_2_ products. Moreover, the coupled metal sites in DACs can make effort on breaking the scaling relationship in the reactions containing multiple proton and electron transfer. DACs have more flexible active sites than SACs. Taking ORR as an example, adsorption configuration of O_2_ could be changed into side-on adsorption on the coupled metal sites whereafter resulting in the formation of two metal–oxygen bonds and elongating the O–O bond. This activation approach takes effect in the breakage of the O–O bond, lowers the energy barrier and partially eliminates the linear relationship [32]. In conclusion, the construction of dual metal active sites is considered as a promising technique to further enhance the activity, especially for multistep reactions.

## Classification of Dual-Atom Catalysts

In SACs, metal sites are isolated and do not have interactions. When the distance (*d*) between the metal sites is shortened to a certain extent, there is an interaction between two close metal atoms that synergistically adjusts the electronic properties of the metal sites [33]. In DACs, metal atoms should be paired and interact with each other in terms of space structure and electronic states [26]. Jin et al. experimentally demonstrated that when the atomic distance between the single-atom Fe sites was reduced to approximately 12 Å, interactions between Fe–N_4_ began to occur, leading to changes in electronic structure and catalytic activity [34]. Tamtaji et al. derived from theoretical calculations that Fe–M acts as a DAC (dual-atom catalyst) when the distance between the Fe atom and another metal atom becomes less than 15 Å [35]. In addition, recent studies on DACs have revealed that metal atoms are already in close proximity to each other when the distance of metal atoms is narrowed to 2 ~ 3 Å by electron microscopy and X-ray spectroscopy measurements [36–41]. Therefore, in this chapter, DACs are classified by the distance (*d*) and connection mode of metal sites: DACs with no contact sites (3 Å < *d* < 15 Å), DACs with metal–metal bonds (2 Å < *d* < 3 Å), DACs with metal sites bridged by nonmetal atoms (2 Å < *d* < 3 Å). The latter two DACs have similar d values, which are generally further determined by the resolution of the fine structure of the metal atoms.

### DACs with No Contact Sites

**Fig. 2:**
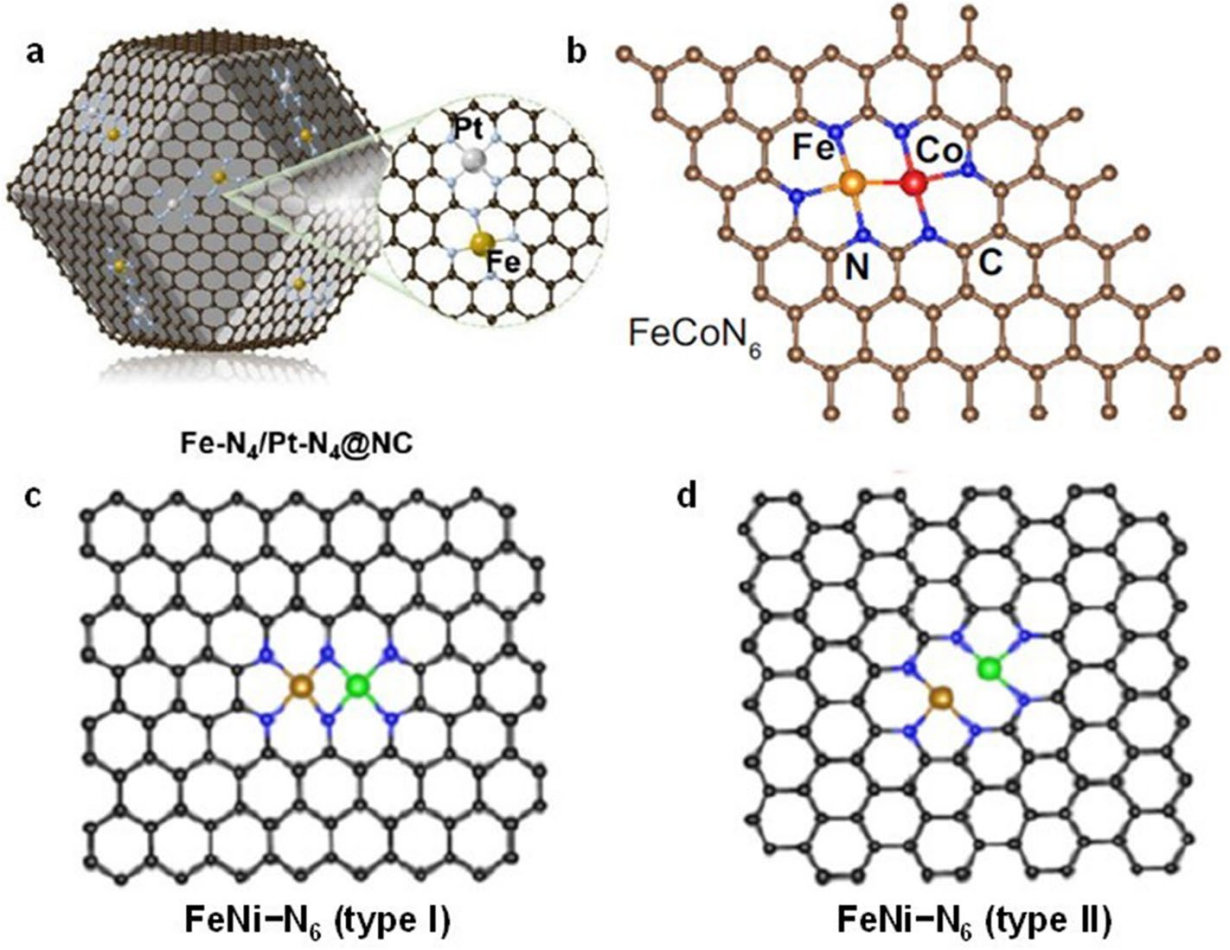
**a** Schematic of Fe–N_4_/Pt–N_4_@NC [42]. Copyright 2021 Wiley–VCH GmbH. **b** Structure of FeCoN_6_ [46]. Copyright 2023 Springer Nature. **c** Structural models of FeNi–N_6_ (type I) and **d** FeNi–N_6_ (type II) [48]. Copyright 2020 American Chemical Society

Although not in contact with each other, when the atomically dispersed metal sites are spaced sufficiently small, interactions can occur to obtain the desired catalytic performance. Therefore, DACs with single-atomic sites close together but not next to each other have been developed (Fig. 1b). Han et al. found that the adjacent Pt–N_4_ site can effectively modulate the 3*d* electron orbitals of the single-atom Fe–N_4_ site by density functional theory (DFT) calculations (Fig. 2a) [42]. They obtained Fe–N_4_/Pt–N_4_@NC by pyrolysis of the ZIF-8 encapsulated with Fe species and Pt species. Fe–N_4_/Pt–N_4_@NC showed catalytic activity in ORR that far exceeds that of single-atom Fe–N_4_@NC, demonstrating that the adjacent Pt–N_4_ moiety is able to exert a significant electron modulation effect on Fe–N_4_ moiety. Wu et al. successfully prepared DAC with Fe sites and Co sites adjacent to each other (named FeCo-NSC) by a soft template-directed interlayer confinement strategy [43]. The FeCo-NSC has an FeN_4_S_1_–CoN_4_S_1_ structure, in which the Fe and Co sites can synergistically lower the reaction energy barrier to obtain excellent ORR electrocatalytic activity. Hu et al. attempted to improve the selectivity of CO_2_ reduction reaction (CO_2_RR) by introducing hydrogen-evolution-reaction-inert main-group metal single atoms near the single-atom Cu atoms [44]. The prepared Cu-In-NC exhibits excellent CO_2_ electrochemical reduction activity with ultra-high Faraday efficiency for CO products. This dual-atom site construction strategy provides additional opportunities to enhance the catalytic performance.

### DACs with Metal–Metal Bonds

For this kind of DAC, M and M’ are bonded together by metal–metal bond to work synergistically as new active site (Fig. 1c). Zhang et al. found that the construction of DACs with bonded atomic pairs of Fe–Ni sites could overcome the limitations of unilateral selective adsorption and activation of carbon or nitrogen species to achieve efficient electrochemical synthesis of ammonia [45]. The Fe–Ni site can act as active site, activation site, and coupling site at the same time, thus effectively promoting C–N coupling. The prepared diatomic Fe–Ni catalyst achieves a super high urea yield and nearly 100% Faraday efficiency. DACs with Fe–Co sites were developed by Sun et al. to address the slow redox kinetics of polysulfides and the slow decomposition of Li_2_S in lithium-sulphur batteries (Fig. 2b) [46]. They found that charge redistribution in the bonded Fe–Co sites promoted the adsorption of LiPSs, thus significantly enhancing the catalytic performance. Zhang et al. designed a DAC with Ni–Cu sites for the electrochemical reduction of CO_2_ to CO [47]. The prepared Cu/Ni-NC exhibited ultra-high Faraday efficiency (~ 99%) and large partial current densities of CO in acidic, neutral, and alkaline media. The constructed Ni–Cu sites could achieve efficient CO_2_RR in acidic electrolytes, which is expected to avoid the use of neutral or alkaline electrolytes, thus solving the problem of carbonate formation.

### DACs with Metal Sites Bridged by Nonmetal Atoms

In this kind of DACs, M and M’ are bridged by nonmetallic atoms (generally N or O) to form dual-atom active sites instead of direct bonding (Fig. 1d). Despite the close spacing between M and M’, no bonding exists between the metal atoms in such DACs. It is worth mentioning that partially bridged diatomic sites and bonded diatomic sites exhibit similar compositions. For example, FeNi–N_6_ (type I) with the structure shown in Fig. 2c was prepared by Zhou et al. [48]. It is coordinated with four N atoms per metal in the diatomic sites. Furthermore, the structure was also compared with FeNi–N_6_ (type II), which has metal–metal bond in the diatomic site and three N atoms per metal coordination (Fig. 2d). DFT calculation analysis showed that the catalytic activity of FeNi–N_6_ (type I) was superior to that of FeNi–N_6_ (type II) in ORR, revealing the importance of the structural configuration of the diatomic sites. Bai et al. prepared a Co–Fe double-atom catalyst for the oxygen evolution reaction (OER) [49]. Co–Fe sites were obtained by in situ electrochemical activation of Co single-atom catalyst. They prepared Co–Fe–N–C in which Co and Fe atoms bridged with two O atoms, exhibiting an ultra-high turnover frequency (TOF) and good stability in the OER. Fan et al. designed an In–Ni-based DAC with an active site configuration of O–In–N_6_–Ni moiety for high-efficiency CO_2_RR [50]. In the O–In–N_6_–Ni moiety, In and Ni atoms are bridged by an axial O atom in addition to co-coordination of two N atoms. In situ attenuated total reflection surface-enhanced infrared absorption spectroscopy (ATR-SEIRAS) and DFT calculations reveal that the In-Ni site acts synergistically with the O bridge to reduce the formation energy barrier of *COOH in CO_2_RR and prevent HER onset, resulting in a high Faraday efficiency and large CO partial current density. Besides the above, Gong et al. designed a highly active ORR catalyst consisting of a single O atom bridging two FeN_4_ parts to form the active site [51]. This unique OFeN_4_–O–FeN_4_O site exhibited a TOF that far exceeded of the FeN_4_ site and was proved to be more stable by DFT calculations.

## Dual Metal Sites Synergistic Effect

DAC is an important branch of atomically dispersed catalysts, whose synergistic effect is thought to be able to compensate for many of the deficiencies of traditional SACs. They not only retain the advantages of SACs, but also offer more opportunities to regulate the active site when a second atom is introduced. The two adjacent metal active centres may play different roles and synergistically alter catalytic behavior [52]. At present, the research on DACs covers two aspects of homonuclear DACs and heteronuclear DACs [31, 37, 53–60]. The synergistic effect of them would be overview in this chapter.

### Synergistic Effect in Homonuclear DACs

At an early stage, the study of DACs was mainly about homonuclear DACs. Chen et al. studied the acetoxylation of ethylene to vinyl acetate (VA) on a palladium (Pd)–Au alloy catalyst [61]. They found that low Pd coverages were more effective for VA synthesis. Comparison of catalysts with different Pd coverage demonstrated that a pair of Pd monomer is superior to a single isolated Pd site. Subsequently, different types of homonuclear DACs were developed and extended to other catalytic fields. The synergistic mechanism of homonuclear DACs has been extensively studied.

#### Optimization on the Adsorption Configurations of Reactants and Intermediates

The adsorption configurations of reactants and intermediate states are directly related to the catalytic performance. According to Sabatier principle, either too strong or too weak adsorption leads to more sluggish reaction processes [62]. In order to visually compare the active sites, the adsorption free energy was used as a descriptor of the catalytic activity to plot volcano diagram. The introduction of a second metal site near the single-atom site to form DAC is expected to push the related adsorption free energy towards the volcano apex and thus achieve a more satisfactory catalytic activity.

**Fig. 3:**
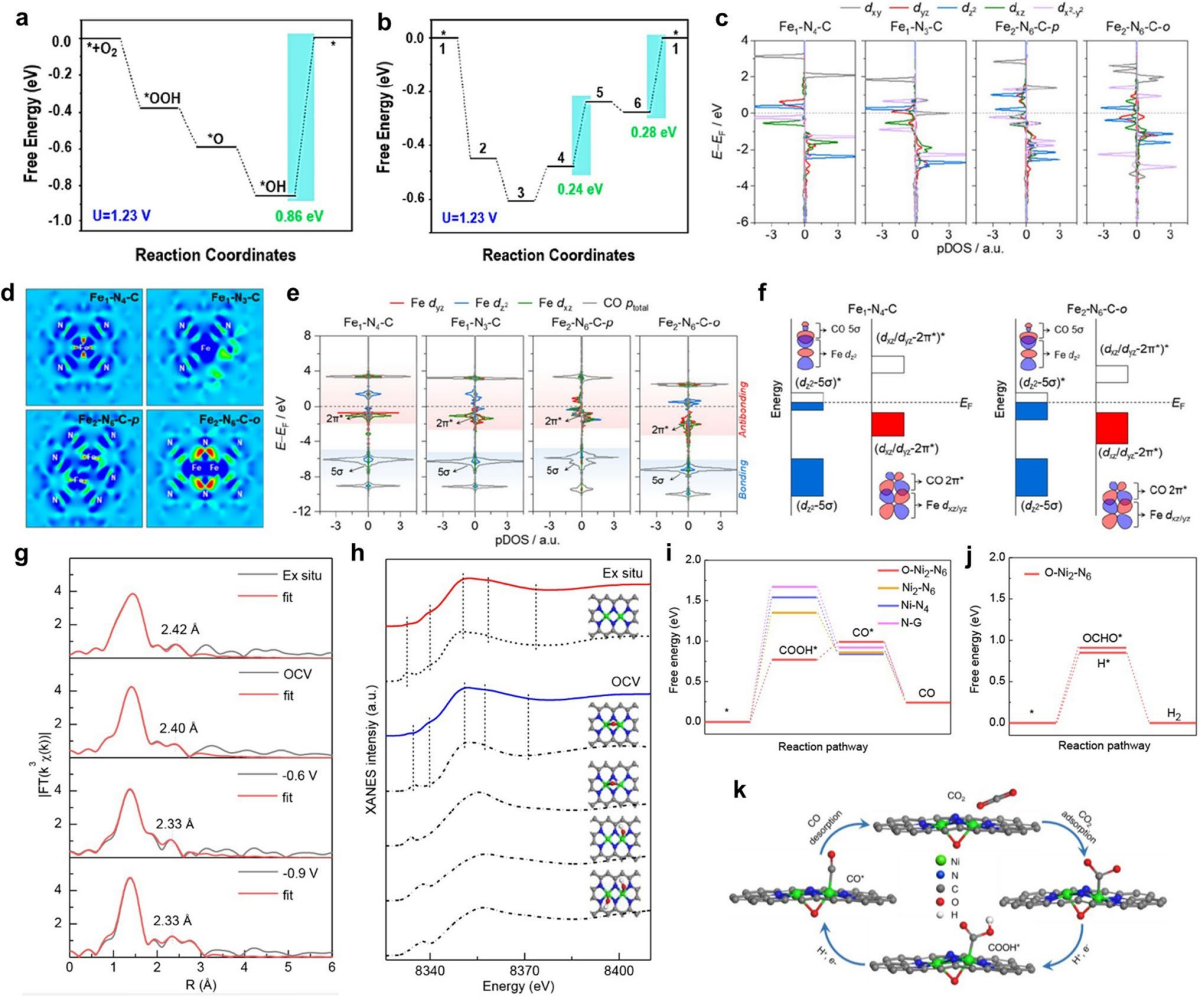
**a** Free energy diagram for ORR on a single Fe site of molecular FePc. **b** Free energy diagram for ORR on two adjacent Fe sites of FePc nanorods [63]. Copyright 2022 American Chemical Society. **c** Partial density of state (pDOS) of Fe 3*d* orbitals. **d** Charge density difference. **e** pDOS for Fe-3*d*_yz_/*d*_z_^2^/*d*_xz_ orbitals and adsorbed CO orbitals. **f** Orbital interaction between Fe-3*d* (*d*_z_^2^ and *d*_xz_/*d*_yz_) and adsorbed CO (5σ and 2π*) [30]. Copyright 2022 American Chemical Society. **g** Least-squares curve-fitting analysis of operando EXAFS spectra at the Ni K-edge. **h** Comparison between the Ni K-edge XANES experimental spectra (solid lines) and the theoretical spectra (dashed lines) calculated with the depicted structures (insert). **i** Free energy diagrams for CO_2_ electroreduction to CO. **j** Calculated Gibbs free energy diagrams for the HER and CO_2_ electroreduction to HCOOH on O − Ni_2_ − N_6_. **k** Proposed reaction pathways on O − Ni_2_ − N_6_ [37]. Copyright 2022 American Chemical Society

Different from SACs with random locations, Kumar et al. reported that FePc nanorods with diatomic Fe sites were synthesized by face-to-face assembly of molecular phthalocyanine [63]. The robust activity was attributed to the specific adsorption configuration of oxygen molecules on the dual metal Fe sites. The free energy diagram revealed the presence of OH* over adsorption on the single Fe sites (Fig. 3a). After the construction of dual-atom Fe sites, the reaction path is optimized and the overpotential is reduced to 0.28 eV, effectively improving the reaction activity (Fig. 3b). Tian et al. selected (Ethylenediamine)iodoplatinum(II) dimer dinitrate as diatomic Pt precursor and mesoporous graphitic carbon nitride (mpg-C_3_N_4_) as the substrate [64]. The prepared Pt_2_/mpg-C_3_N_4_ showed remarkable catalytic ability towards the selective hydrogenation of nitrobenzene to aniline. The interaction between Pt atoms and oxygen atoms leads to the easy fracture of N–O bonds, resulting in unsaturated N and O adsorbates. The unsaturated N and O are conducive to H_2_ dissociation and achieve high efficiency reaction of nitrobenzene hydrogenation to aniline. Quan et al. achieved efficient electrochemical reduction of CO_2_ by constructing homonuclear diatomic Fe–Fe sites on nitrogen-doped carbon substrates [65]. DFT simulations show that the adsorption energy of *CO_2_ on diatomic Fe–Fe sites is much lower than that on single-atom Fe sites, which is the key to the enhancement of catalytic activity. The mechanism of this reduced adsorption energy originates from the bridge-like adsorption of CO_2_ molecules on diatomic Fe–Fe sites.

#### Alteration of the Charge Configuration of the Active Site

The charge configuration of active site can directly affect the adsorption and desorption of reactants, thus changing the reaction energy barrier. The superior catalytic performance of SACs has been achieved by adjusting charge configurations [66–69]. Among them, the introduction of a second metal atom to construct a dual metal pair active site has been proved as a promising way to realized desired charge configurations for various reactions [70, 71]. For example, Wang et al. prepared atomically dispersed Fe-based catalysts by adjusting the pyrolysis scheme of Fe-ZIF-8 precursor [30]. The Fe_2_–N–C DAC prepared under H_2_ pyrolysis condition exhibited excellent Faradaic efficiency above 80% and higher turnover frequency in CO_2_ electroreduction. DFT calculations were carried out to clarify the synergistic effect of Fe pair in Fe_2_–N–C DAC. Partial densities of state (pDOS) demonstrated that Fe-3*d* orbitals were delocalized in the paired Fe sites, leading to the decrease of orbital energy levels and delocalization of electrons (Fig. 3c). Therefore, the *CO was easier to desorb on Fe_2_–N–C DAC. The charge density difference plots suggested that more electrons were transferred from Fe to the coordinated N atom in Fe_2_–N_6_–C-*o* (Fig. 3d). Further calculation of *CO adsorption configuration indicated that the charge transfer on the Fe_2_–N_6_–C-*o* was the least and the energy gap between antibonding and bonding states was much smaller (Fig. 3e, f). The synergistic effect above endowed Fe_2_–N–C-*o* remarkable catalytic performance. Wang et al. prepared supramolecular precursors from melamine, cyanuric acid and Co salt [54]. The precursors were further pyrolyzed to produce atomic dispersed Co-based catalysts for CO_2_ photoreduction. Co-based homonuclear DACs could be obtained by adjusting the amount of Co salt. Among them, CoDAC-3.5 showed a superior CO_2_RR performance than Co-based SACs. Different from Co-based SACs, electrons are homogeneously enriched around Co_2_ sites. This change in charge configuration allowed the rate-limiting COOH* intermediate to stabilize more efficiently and reduces the high Gibbs free energy of forming COOH* (ΔG(COOH*).

#### Evolution of More Favourable Active Site

At present, the exact reaction mechanism on DACs remains open questions. The key to identify the catalytic mechanism of DAC is to recognize the electron and structure evolution of DACs at the atomic level under real reaction conditions.

Ding et al. successfully prepared a precise DAC Ni_2_/NC by ligand protection strategy [37]. The as-prepared Ni_2_/NC exhibited a CO formation Faradaic efficiency of 94.3% towards CO_2_RR. Moreover, this work employed *operando* X-ray absorption fine structure (XAFS) technique to explore the structural evolution of the dinuclear Ni_2_ sites under real reaction conditions. Specifically, they found that the catalyst can adsorb oxygen-relevant intermediates to form a configuration of O–Ni_2_–N_6_ under the open-circuit condition (Fig. 3g). After the formation of O–Ni_2_–N_6_ site, the atomic spacing of Ni_2_ pairs is shortened by 0.2 Å, which leads to stronger interaction between Ni atoms. The XANES simulation calculations fitting provided additional evidence that the oxygen species coordinated on the Ni_2_–N_6_ site was bridge oxygen (Fig. 3h). Further DFT calculations demonstrated that O–Ni_2_–N_6_ site displayed more suitable ΔG and lower energy barrier forming COOH* (Fig. 3i). Moreover, the Gibbs free energy of hydrogen adsorption and the first step forming OCHO* were both higher than that of COOH* on O–Ni_2_–N_6_ site, resulting in high selectivity of CO production (Fig. 3j, k). Hao et al. explored a Ni-based homonuclear DAC via in situ conversion of nanoparticles into dual-atom sites [72]. Ni atoms undergone Ostwald ripening and atomization on the defective carbon to form the Ni_2_N_6_ sites. The as-prepared Ni DAC exhibited nearly 100% Faradaic efficiency towards CO production and high current density up to ~ 1 A cm^−2^ in CO_2_RR. In situ X-ray absorption suggested that the Ni dual-atom sites could adsorb hydroxyl (OH_ad_) in solution first, forming the unique electron-rich Ni_2_N_6_OH structure. Theoretical calculation proved that the construction of this electron-rich centre can effectively optimize the adsorption of *COOH and the desorption of *CO, thus reducing the kinetic energy barrier of the whole reaction.

### Synergistic Effect in Heteronuclear DACs

Compared with the homonuclear DACs, the heteronuclear DACs are featured by the combination of two different metal atoms as active centres. Heteronuclear DACs are considered to have greater research potential compared with homonuclear DACs since different metal atoms can combine into pairs, leading to more flexible structures. Therefore, more types of feasible electronic structure can be developed to create diverse synergetic effects in heteronuclear DACs towards to different reactions. In heteronuclear DACs, two different metal atoms result in asymmetric active sites and electronic structure, which makes metal sites more active. The synergistic effect between metal sites in DACs would lead to a change in the d-band centre and significantly improved catalytic performance [73]. As a result, more and more researchers have focused on the study of heteronuclear DACs [74–80]. However, there are still a lot of mysterious issues about the synergistic effects of heteronuclear DACs in different reactions.

#### Redistribution of Charge

**Fig. 4:**
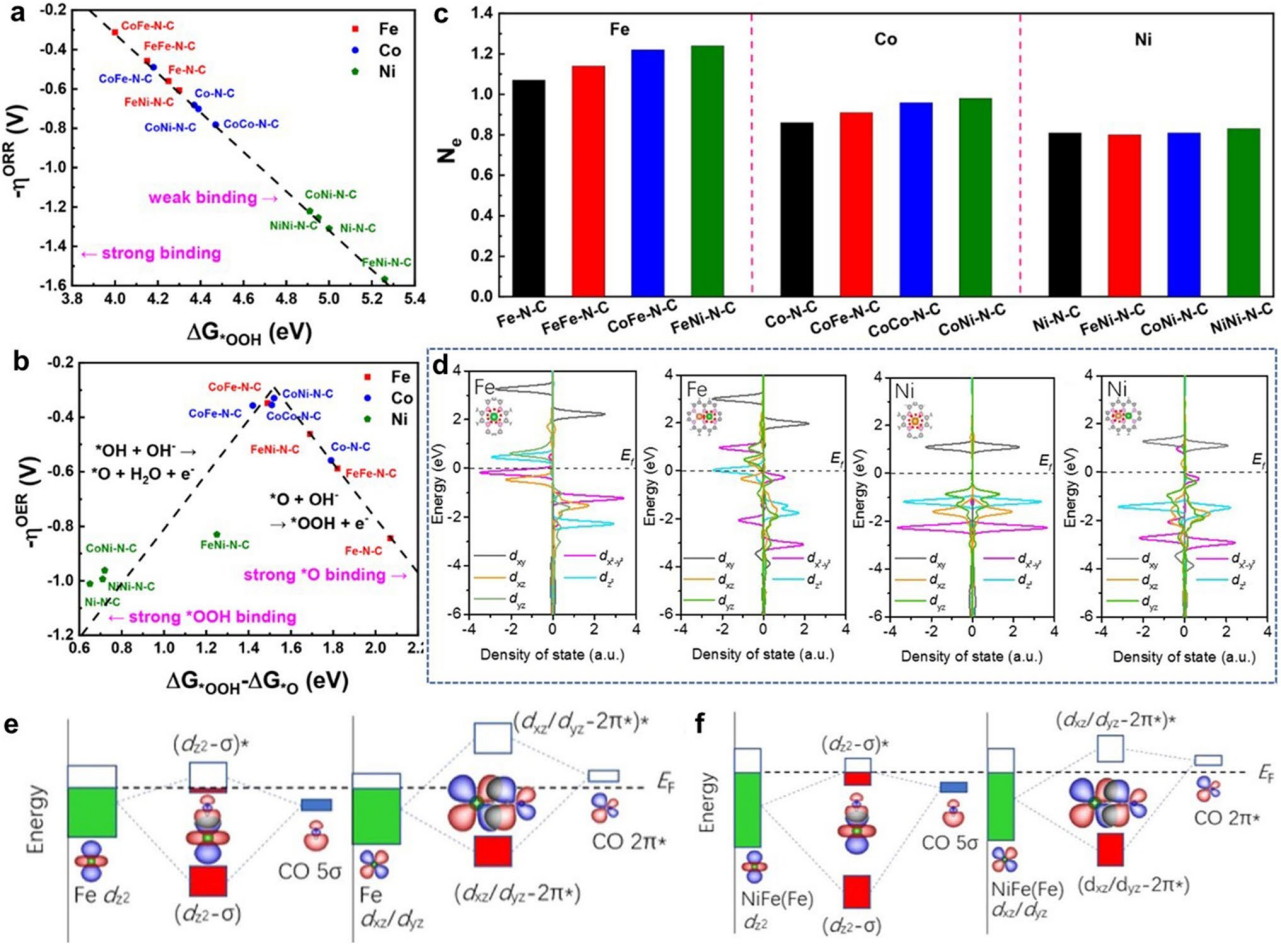
**a** ORR theoretical overpotential (η^ORR^) versus ΔG_*OOH_. **b** Volcano plots of the OER theoretical overpotential (η^OER^) versus adsorption free energy difference (ΔG_*OOH_—ΔG_*O_). **c** Number of electrons (N_e_) lost from an Fe, Co, or Ni atom [81]. Copyright 2022 American Chemical Society. **d** Density of states of Fe 3*d* for Fe-SAC and NiFe-DASC, of Ni 3*d* for Ni-SAC and NiFe-DASC. **e** Schematic illustration of orbital interactions between adsorbed CO (5σ and 2π*) and 3*d* orbital (*d*_z_^2^, *d*_xz_/*d*_yz_) of Fe site in **e** Fe-SAC and **f** NiFe-DASC [83]. Copyright 2021 Springer Nature

Heteronuclear DACs change the charge distribution of metals by constructing atomic pairs of different metal atoms, which is unreachable for homonuclear DACs. Heteronuclear DACs can optimize the adsorption of reaction intermediates and reduce the energy barrier by regulating the charge density of the active site reasonably. Zhou et al. used DFT simulation to screen the best DAC composed of transition metals Fe, Co, and Ni [81]. To identify the catalytic ability of these DACs in ORR and OER, their performance in the potential-determining step (PDS) was compared separately (Fig. 4a, b). Among these models, the CoFe–N–C could promote both ORR and OER for efficient bifunctional catalysis. The Bader charge analysis shows that the construction of dual metal sites leads to tunable charge distributions, which is favourable for the modulation of the catalytic activity (Fig. 4c). Due to the different electronegativity between metal atoms, the degree of charge transfer between heteronuclear diatomic pairs is quite distinct. Hao et al. developed a DAC with Cu and Ni bimetal sites supported on electrospun carbon nanofibers (CuNi-DSA/CNFs) [82]. The electronegativity compensation between Cu and Ni bimetal sites results in strong electron interactions. Bader charge distribution shows that Ni and Cu sites in CuN_4_–NiN_4_ have more electrons compared to corresponding single-atom sites. In addition, the projected density of states (PDOS) also demonstrated the presence of active electron exchange between Cu and Ni sites. The free energy diagram of the CO_2_RR process further indicated that the electronegativity compensation effect obviously lowers the energy barrier of CO_2_ adsorption and *COOH formation which is the rate-determining step (RDS) of CO production. The CuNi-DSA/CNFs delivered an ultrahigh Faradic efficiency (99.6%) of electrochemical reduction of CO_2_ to CO, which was consistent with the theoretical analysis above.

#### Regulation of *d* Orbitals of Metal Sites

The active sites of DACs studied so far are mainly composed of transition metal atoms. The synergistic effect can be achieved by modulating the electronic structure of the d orbitals of the transition metal sites to achieve different catalytic properties. Interactions among the d orbitals in the transition metal pairs can modulate the orbital energy levels, thus changing the adsorption of the reaction intermediates. Zeng et al. explored a bifunctional catalyst for CO_2_RR and OER with Ni and Fe diatomic sites (denoted as NiFe-DASC) [83]. NiFe-DASC exhibited catalytic activity and durability far superior to corresponding Ni-SAC and Fe-SAC. In-depth electronic structure analysis elucidates the origin of the catalytic performance of NiFe-DASC. DFT calculations revealed that the *d*_xz_ and *d*_yz_ orbitals of Fe in NiFe-DASC exhibited a lower degree of localization compared with FeSAC, while the *d*_z_^2^ orbital state of Ni crossed the Fermi energy level (Fig. 4d). Further analysis indicated strong d-d orbital coupling between Ni and Fe atoms. The energy levels of the *d*_z_^2^, *d*_x_^2^_−y_^2^, *d*_xz_, *d*_xy-_*p*_z_*p*_y_ and *d*_yz_-*p*_x_ orbitals changed significantly. Among them, *d*_z_^2^, *d*_x_^2^_−y_^2^ and *d*_xz_ interact with each other, leading to the decrease of the orbital energy level and electron delocalization, which favours the desorption of *CO. Moreover, the Fe sites in NiFeDASC exhibited decreased energy levels of bonding and antibonding states, resulting in larger electron occupancy (Fig. 4e, f). Kong et al. proposed a d-orbits symmetry modulation strategy for efficient oxygen reduction in acidic media [84]. (Au–Co) DP-NPAs with atomically dispersed Au–Co sites were successfully prepared by pyrolysis Co-doped ZIF-8 hosting tetrachloroaurate precursor. The ORR activity of Au–Co sites outperforms single-atom Au or Co sites a lot. In order to further understand the catalytic behavior of (Au–Co) DP-NPAs, the projected density of states (PDOS) calculation was proposed based on d-orbital configuration and atomic coordination symmetry. When *OH adsorbs on the Co atom, the coordinated symmetry of the adjacent Au atom changes from C_2v_ to C_2_. Therefore, the *d*_x_^2^_−y_^2^ antibond spin-orbital goes down to a lower energy level, leading to a higher ORR activity. Pei et al. synthesized atomically dispersed Ni/Co dual sites immobilized on nitrogen-doped carbon (a-NiCo/NC) by a multi-step template method with an atomic migration capture process [85]. The a-NiCo/NC exhibited a low overpotential of 252 mV at 10 mA cm^−2^ in OER and could work steadily for 150 h. DFT calculations were conducted to reveal the synergistic effect between Ni atoms and Co atoms in a-NiCo/NC. After the formation of Ni/Co diatomic pairs, the PDOS demonstrated a strong electronic coupling between Co and Ni atoms and significant upward shifts of the d-band centres of both Ni and Co to the Fermi level. The increased antibonding states could adjust the electronic structure of Ni and Co atoms in a-NiCo/NC to enhance bonding ability for OER intermediates.

#### Manipulation of Electronic Spin Configurations

**Fig. 5:**
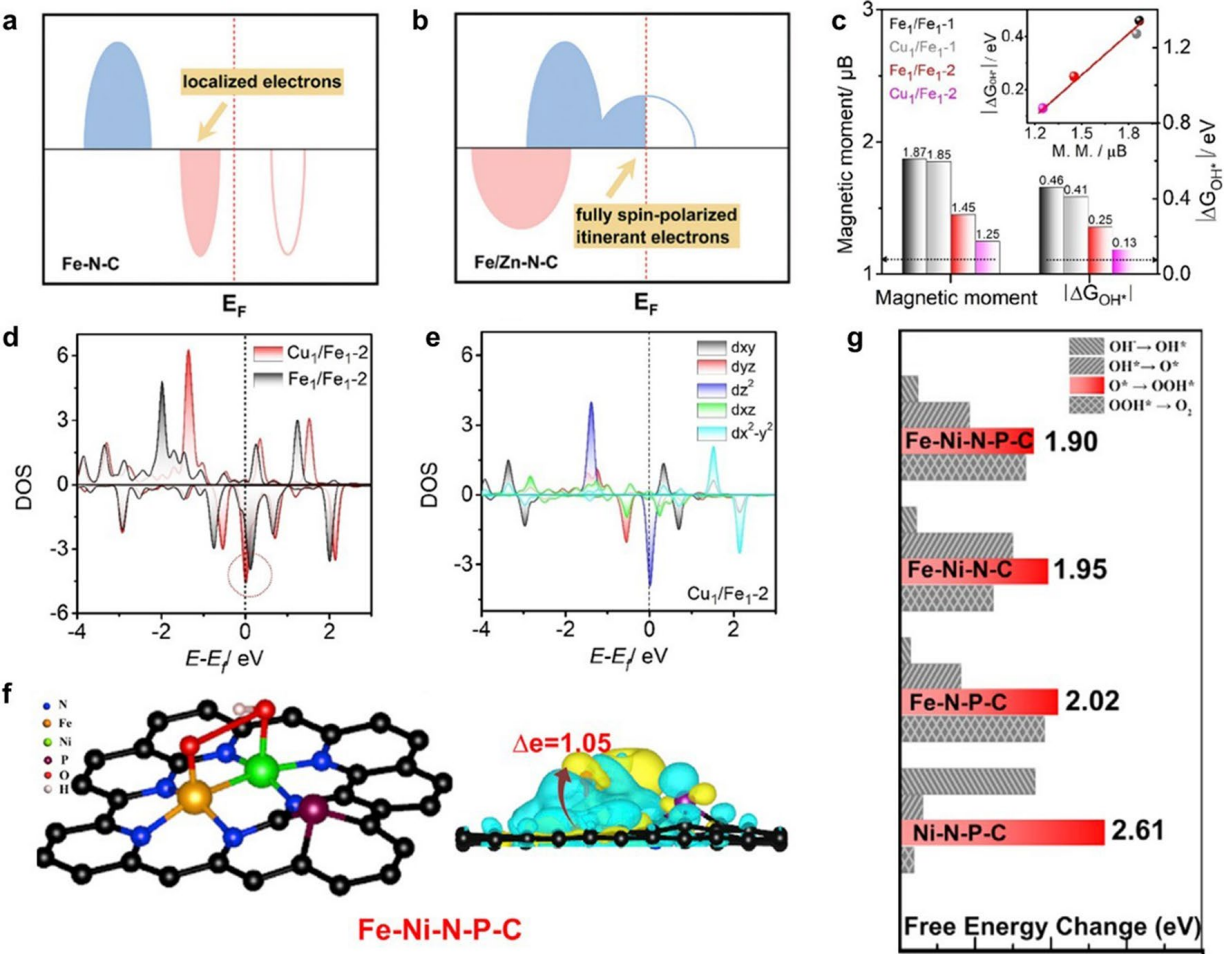
Schematic electronic structures of **a** Fe–N-C and **b** Fe/Zn-N–C [88]. Copyright 2022 Royal Society of Chemistry. **c** Comparison of magnetic moment and ΔG_OH*_. **d** Fe 3*d* DOS of Fe_1_/Fe_1_-2 and Cu_1_/Fe_1_-2. **e** DOS of the five Fe 3*d* orbitals in Cu_1_/Fe_1_-2 [89]. Copyright 2022 Wiley–VCH GmbH. **f** Geometric structure and corresponding electron density difference of Fe–Ni–N–P–C. **g** Overpotential of OOH* formation [91]. Copyright 2021 Elsevier Ltd

The spin configuration is thought to be able to greatly influence the catalytic activity of SACs and DACs [68, 86]. Proper modulation of the spin configuration can contribute to optimizing the adsorption of reaction intermediates, thus accelerating the reaction kinetics. Li et al. investigated the charge itineration and electron spin polarization of heteronuclear DAC in bifunctional ORR/OER electrocatalysis by DFT calculations for the first time [87]. Theoretical calculations show that Fe–N–C(OH) exhibits insulating properties, while Ni–N–C(OH) is conductive. Therefore, the construction of Fe–Ni diatomic sites can improve the electrical conductivity of the Fe sites to facilitate the charge transfer process required for the reaction. Moreover, the moderate stray field generated by the spin polarization of transition metals can effectively modulate the adsorption strength of paramagnetic O_2_ molecule. The weak spin magnetization of Ni atoms in Ni–N–C leads to the difficulty of oxygen capture. After the formation of Fe–Ni sites, the active centre possesses mild spin magnetization and finite spin-polarized conduction electrons, which facilitates the generation of stray field to promote O_2_ trapping and O–O bond formation, thus improving the catalytic activity of ORR/OER. The results of the subsequent experimental tests were consistent with the above theoretical study. Li et al. synthesized Fe/Zn–N–C DAC with Fe–Zn dual metal sites after theoretical screening [88]. The Fe/Zn–N–C exhibited excellent half-wave potentials of 0.906 and 0.808 V under alkaline and acidic conditions, respectively. Theoretical studies have shown that single-atom Zn sites constructed near single-atom iron sites are capable of filling spontaneous spin-polarized electrons at the Fermi level leading to a transition of the active centre from semiconductor to semimetal (Fig. 5a, b). Furthermore, the stray field generated by the spin-polarized Fe/Zn–N–C could induce the spin of two unpaired electrons in the O_2_ molecule to orientate in the same direction, thus facilitating the adsorption of O_2_ molecule and the formation of Fe–O bond. To investigate the effect of spin state in ORR, He et al. analysed the relationship between the magnetic moment and desorption energy of –OH (ΔG_OH*_) [89]. They demonstrated the spin magnetic moment of the target DACs could form a good linear relationship with ΔG_OH*_ through theoretical simulations (Fig. 5c). Compared with the homonuclear DACs Fe_1_/Fe_1_-1 and Cu_1_/Cu_1_-1, the heteronuclear DAC Cu_1_/Fe_1_-2 was able to achieve the magnetic moment manipulation more efficiently and features the lowest ΔG_OH*_. In addition, heteronuclear DAC Cu_1_/Fe_1_-2 showed larger contribution to the DOS near the Fermi level (Fig. 5d). The increasement in the charge density of *d*_z_^2^ orbital was found to be the origin of the change in magnetic moment (Fig. 5e).

#### Induction of Different Adsorption Configurations

Like homonuclear DACs, diatomic active sites in heteronuclear DACs can also alter the adsorption configuration of reactants. Wang et al. synthesized a DAC with atomically dispersed Co–Fe sites (denoted as CoFe-NC) [90]. The CoFe-NC showed remarkable ORR performance with a half-wave potential of 0.94 V. To investigate the catalytic mechanism of CoFe-NC DAC in ORR, the adsorption of O_2_ molecule was calculated. The O_2_ molecule tends to adsorb on metal sites with a side on configuration. The stronger interaction between the O_2_ molecule and the Co–Fe dimer sites was confirmed by the elongated distance of the O=O bond, which could enhance the progress of ORR. Pan et al. revealed that the diatomic catalyst Fe–Ni-N-P–C possessed the lowest energy barrier for OOH* formation of OER by theoretical calculations [91]. Theoretical studies showed that the bidentate binding between two oxygen atoms of OOH* (Fig. 5f) and the dual metal Fe–Ni site achieved a much lower energy barrier of the OOH* formation (Fig. 5g), resulting in a higher ORR catalytic activity.

## Synthetic Methods of DACs

### Homonuclear DACs

**Fig. 6:**
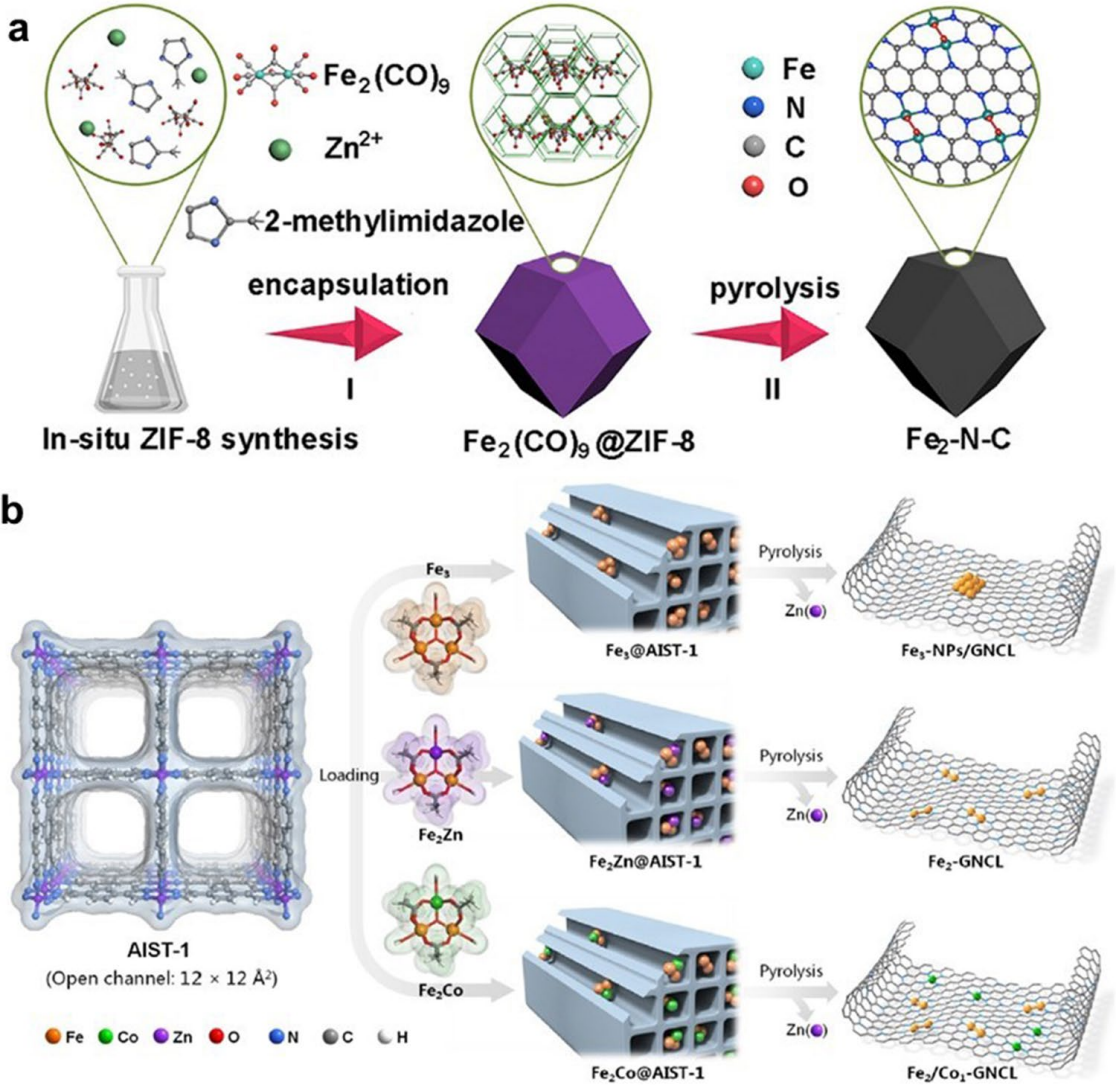
**a** Schematic illustration for the two-step synthesis of Fe_2_–N–C [93]. Copyright 2019 Elsevier Ltd. **b** Heteroatom modulator approach for fabricating dual-atom iron catalysts on carbon layer [95]. Copyright 2020 Wiley–VCH GmbH

DACs, a further development product of SACs, also face the problem of difficulty in precisely controlling the atomic-level dispersion of sites in the synthesis process. The atomically dispersed metal atoms own high surface free energy and tend to agglomerate into nanoparticles. Therefore, the synthesis of DACs requires strong anchoring of isolated metal dimers to prevent aggregation. At present, the synthetic methods are mainly about using coordination atoms to construct strong bonds to anchor metal dimers. Moreover, the synthesis of DACs faces greater challenges due to the precise structure. The precise construction of different types of metal dimers requires more efficient methods of synthesis. In the construction of metal dimers, too strong metal–metal interactions should be prevented from further agglomerating to form clusters or nanoparticles, while insufficient metal–metal interactions may lead to the formation of too many single-atom sites. In order to solve the problems above, it is necessary to further consider the metal–support interactions to reduce the surface free energy and improve the kinetic barriers of atomic diffusion and aggregation [92]. The approach of “precursor pre-selection” was used to precisely regulate the number of metal atoms to prepare DACs. Ye et al. prepared a series of clusters with different numbers of Fe atom anchored on nitrogen-doped carbon by the precursor pre-selection strategy [93]. The fabrication procedure of DAC with Fe_2_ sites is shown as an example in Fig. 6a. Fe_2_(Co)_9_ compound with binuclear Fe atom was selected as precursors to obtain a Fe-based DAC. During the preparation of the Zeolitic Imidazolate Framework (ZIF-8), Fe_2_(Co)_9_ compound was in situ encapsulated in the cavity of ZIF-8 to form the precursor Fe_2_(CO)_9_@ZIF-8. In the subsequent pyrolysis step, Zn atoms in ZIF-8 can be evaporated away and Fe_2_(CO)_9_ compound will be decomposed into Fe_2_ clusters. The large number of separated cavities in ZIF-8 can effectively prevent the agglomeration of Fe atoms during the pyrolysis process. After the pyrolysis process, nitrogen-doped carbon formed during the pyrolysis of ZIF-8 can effectively anchor Fe_2_ clusters to obtain atomically dispersed Fe_2_–N–C DAC. Tian et al. also prepared a DAC with dispersed Fe_2_ clusters loaded on mesoporous carbon nitrides (mpg-C_3_N_4_) through the strategy of precursor pre-selection [94]. The mpg-C_3_N_4_ was synthesized to act as a substrate, and the Fe_2_O_4_C_14_H_10_ was selected as binuclear metal source. There are a large number of sites in mpg-C_3_N_4_ that can anchor Fe_2_O_4_C_14_H_10_ compounds to form a precursor with Fe_2_ species. The subsequent adjustment of the pyrolysis temperature of the precursor allowed the removal of organic ligands and prevents the agglomeration of Fe atoms. The obtained Fe_2_/mpg-C_3_N_4_ DAC showed excellent catalytic properties for alkene epoxidation. Moreover, the general applicability of the method was further proved by synthesizing DACs with Pd_2_ and Ir_2_ clusters.

To further prevent the agglomeration of metal atoms, Wei et al. further developed heteroatom modulator strategies based on preselection of precursors [95]. The heteroatom modulator strategy requires trinuclear metal clusters as precursors, which are nucleated with two target atoms and one heteroatom. As shown in Fig. 6b, a MOF named AIST-1 with rich and orderly aromatic ring array in structure was utilized to capture metal clusters. The AIST-1 can be transformed into a nitrogen-doped carbon layer by pyrolysis treatment, which is regarded as an ideal substrate for the immobilization of Fe clusters. The trinuclear Fe^III^_2_Fe^II^ complex is selected as precursor and encapsulated in the channel of the AIST-1. During the subsequent pyrolysis, the trinuclear Fe^III^_2_Fe^II^ complex decomposes into Fe_3_ trimers. The unstable Fe_3_ trimers tend to agglomerate via the Ostwald ripening process. To prevent metal atoms from aggregating to obtain the target DAC, Fe^II^ is atomic replaced by other metal(II) ions (Zn^II^/Co^II^). Since metal dimers are thermodynamically stable, the two Fe atoms in the precursor form a binuclear site, while the other heteroatom separates out to form the corresponding mononuclear site after the pyrolysis. In particular, Zn atoms could be removed by evaporation at high temperatures to form pure DAC.

**Fig. 7:**
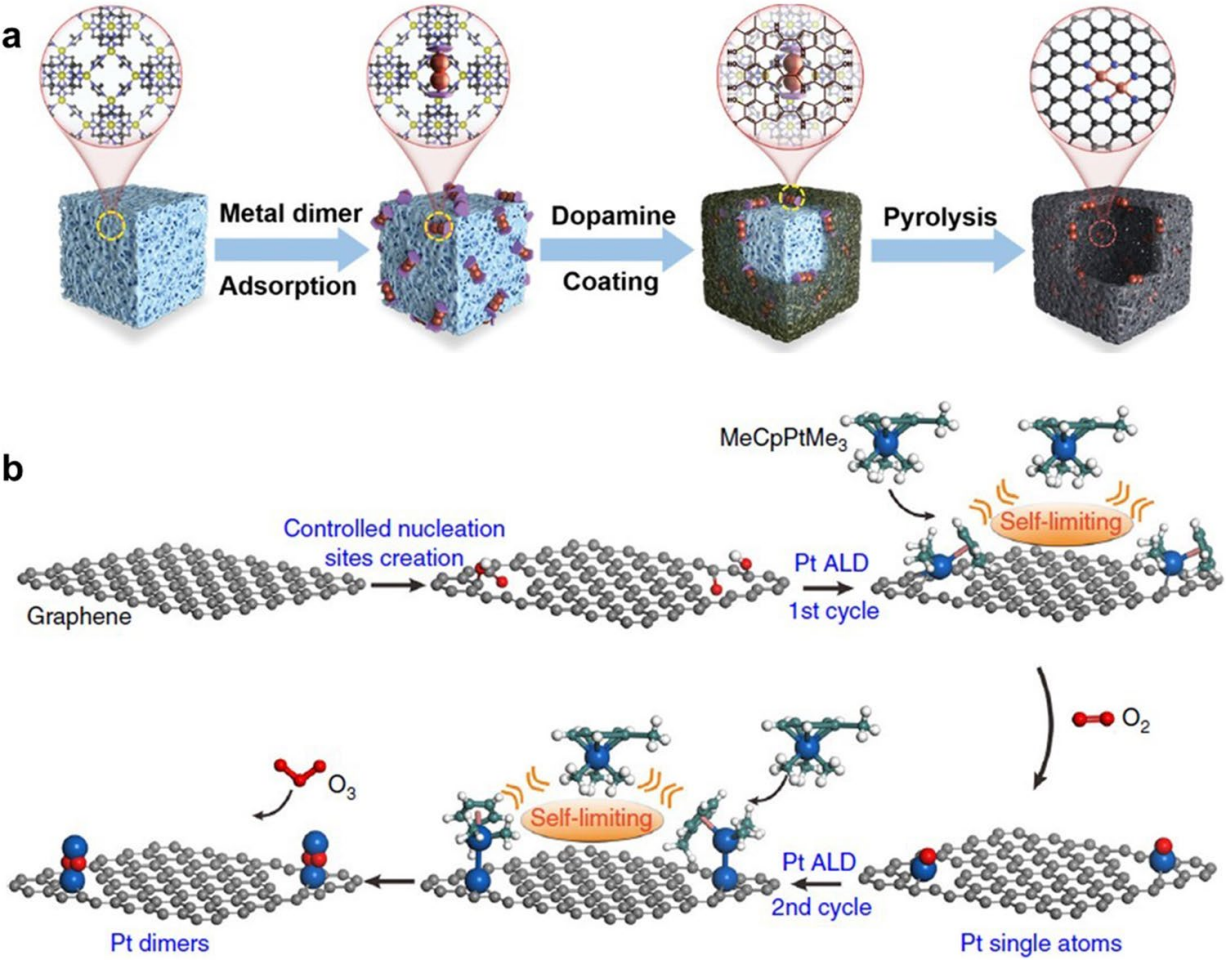
**a** Schematic illustration for the preparation of DACs [96]. Copyright 2022 Wiley–VCH GmbH. **b** Synthesis diagram of Pt_2_/graphene [97]. Copyright 2017 Springer

In order to suppress thermal migration of atoms to obtain stable DACs, Qu et al. proposed an interfacial cladding engineering strategy [96]. As shown in Fig. 7a, a cetyltrimethylammonium bromide (CTAB)-functionalized ZIF-8 was first prepared as the support for metal atoms. The cyclopentadienyliron dicarbonyl dimer (Fe_2_ dimer) was subsequently immobilized on the surface of the (CTAB)-functionalized ZIF-8 by an impregnation–adsorption procedure. After immobilization of Fe_2_ dimer, dopamine polymerizes on the ZIF-8 surface to form a coating layer to encapsulate the Fe dimer. This interfacial cladding engineering prevents Fe_2_ dimer aggregation during the final pyrolysis step and protects the Fe_2_ structure from disruption to form stable DAC with Fe_2_ binuclear sites. Furthermore, DACs containing Cu_2_ and Ir_2_ binuclear sites were also successfully synthesized by the interfacial cladding engineering to verify the universality of the strategy.

Atomic layer deposition (ALD) technology is considered as a potential tool for DACs synthesis because it can be controlled at the atomic scale. Yan et al. successfully synthesized a DAC with Pt_2_ sites through a “bottom-up” method by ALD technology as shown in Fig. 7b [97]. To deposit Pt atoms, graphene with large specific surface area was treated by acid oxidation to form isolated phenols or phenol–carbonyl pairs on the surface as nucleation sites. The first Pt atom was deposited on graphene by alternately exposing trimethyl(methylcyclopentadienyl)-platinum (IV) (MeCpPtMe_3_) and molecular O_2_ at 250 °C to obtain Pt-based SAC (Pt_1_/graphene). The self-limiting surface reaction between MeCpPtMe_3_ and the support ensured that only a single Pt atom was deposited on each site. During subsequent O_2_ exposure process, the ligands could be removed and the individual Pt atoms were exposed. The individual Pt atoms could act as new sites to deposit another Pt atoms to form Pt-based DAC (Pt_2_/graphene). The steric hindrance between MeCpPtMe_3_ molecules prevents further deposition of excess Pt atoms to guarantee accurate synthesis of the Pt_2_/graphene. Finally, Pt_2_ diatomic sites were obtained by removing the surface ligands with strong oxidant treatment. Therefore, ALD is capable to construct catalytic sites uniformly and accurately through the bottom-up pathway, which is favourable for the synthesis of atomic dispersed catalyst.

### Heteronuclear DACs

**Fig. 8:**
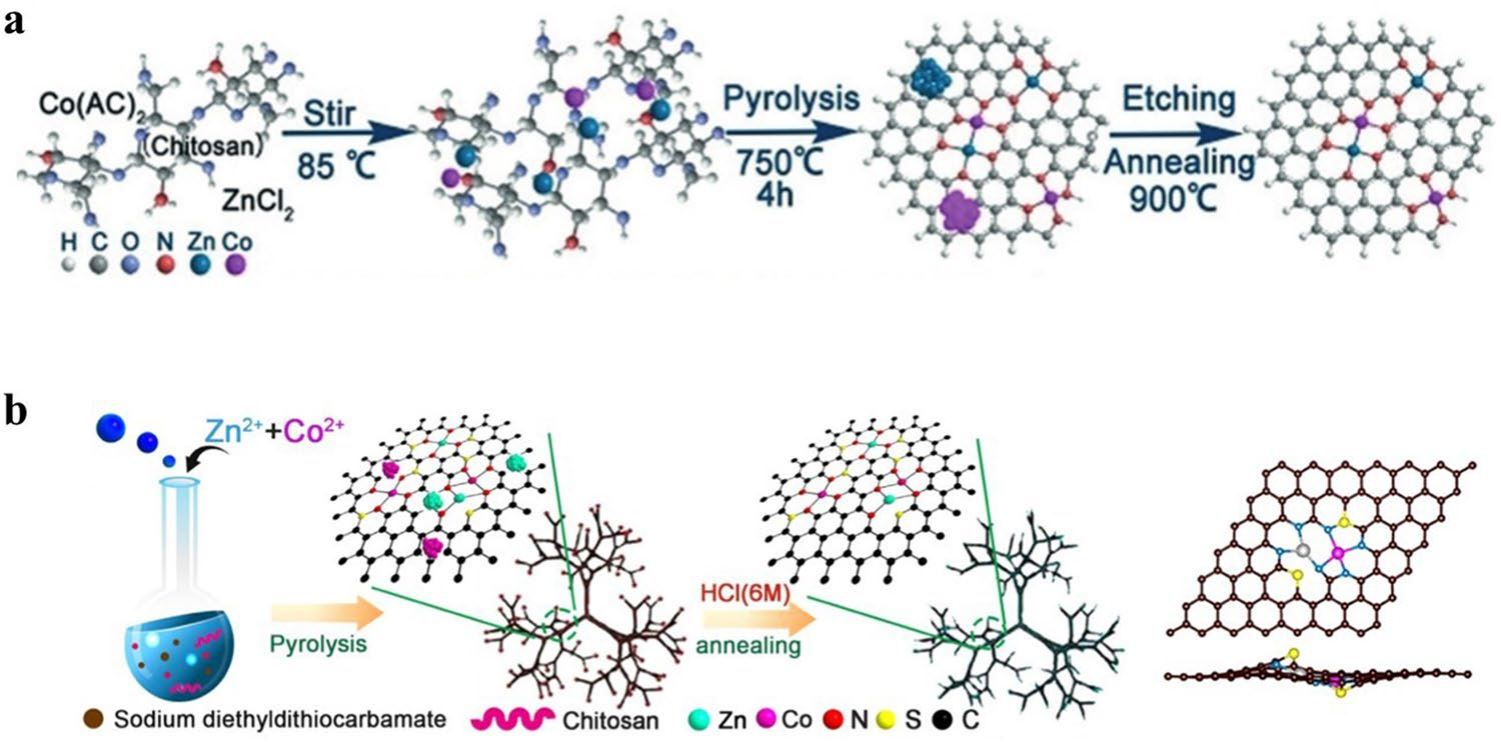
**a** Synthetic diagram of Zn/CoN–C [74]. Copyright 2019 Wiley–VCH GmbH. **b** Schematic diagram of the (Zn, Co)/NSC [102]. Copyright 2019 Elsevier Ltd

Since the synthesis of DACs is more difficult than that of SACs, effective procedures to regulate the formation of dual-atomic sites are highly desired. Heteronuclear DACs can be combined in more ways than homonuclear DACs, which opens up more possibilities for DAC applications [98]. Researchers have done extensive investigations on the synthesis of heteronuclear DACs [99, 100]. Typically, one-pot synthesis strategies are widely used for the synthesis of DACs. Some precursors rich in C, N, and O are coordinated with metal atoms to achieve anchoring during the synthesis process [101]. For example, Lu et al. prepared a DAC named Zn/CoN–C by means of competitive coordination [74]. As shown in Fig. 8a, chitosan was chosen as the carbon and nitrogen source, with zinc chloride and cobalt acetate as the metal source. Chitosan with –NH_2_ and –OH groups has similar coordination ability. The Co^2+^ or Zn^2+^ could coordinate with the –NH_2_ and –OH groups by simultaneous competitive complexation process. The competitive complexation processes enabled metals to form uniformly distributed Co/Zn complexes. After high-temperature pyrolysis the excess metal particles were removed by acid etching to obtain the final DAC with ZnCo binuclear sites. Similarly, Liu et al. prepared a DAC with Zn,Co–N_*x*_–C–Sy active sites limited in dendritic carbon substrate by a simple method of simultaneous coordination pyrolysis [102]. As shown in Fig. 8b, chitosan could also act as a carbon source and provide amino-groups for the simultaneous coordination of Zn^2+^ and Co^2+^ ions. In addition, sodium diethyldithiocarbamate (DDTC) was introduced as the sulphur source. The dissolution of DDTC resulted in the solution transforming into alkaline. This transformation promoted the ethylation and carboxylation of chitosan with DDTC, resulting in a three-dimensional dendritic morphology. After further pyrolysis and acid etching steps, the (Zn,Co)/NSC DAC with S-modification was generated.

**Fig. 9:**
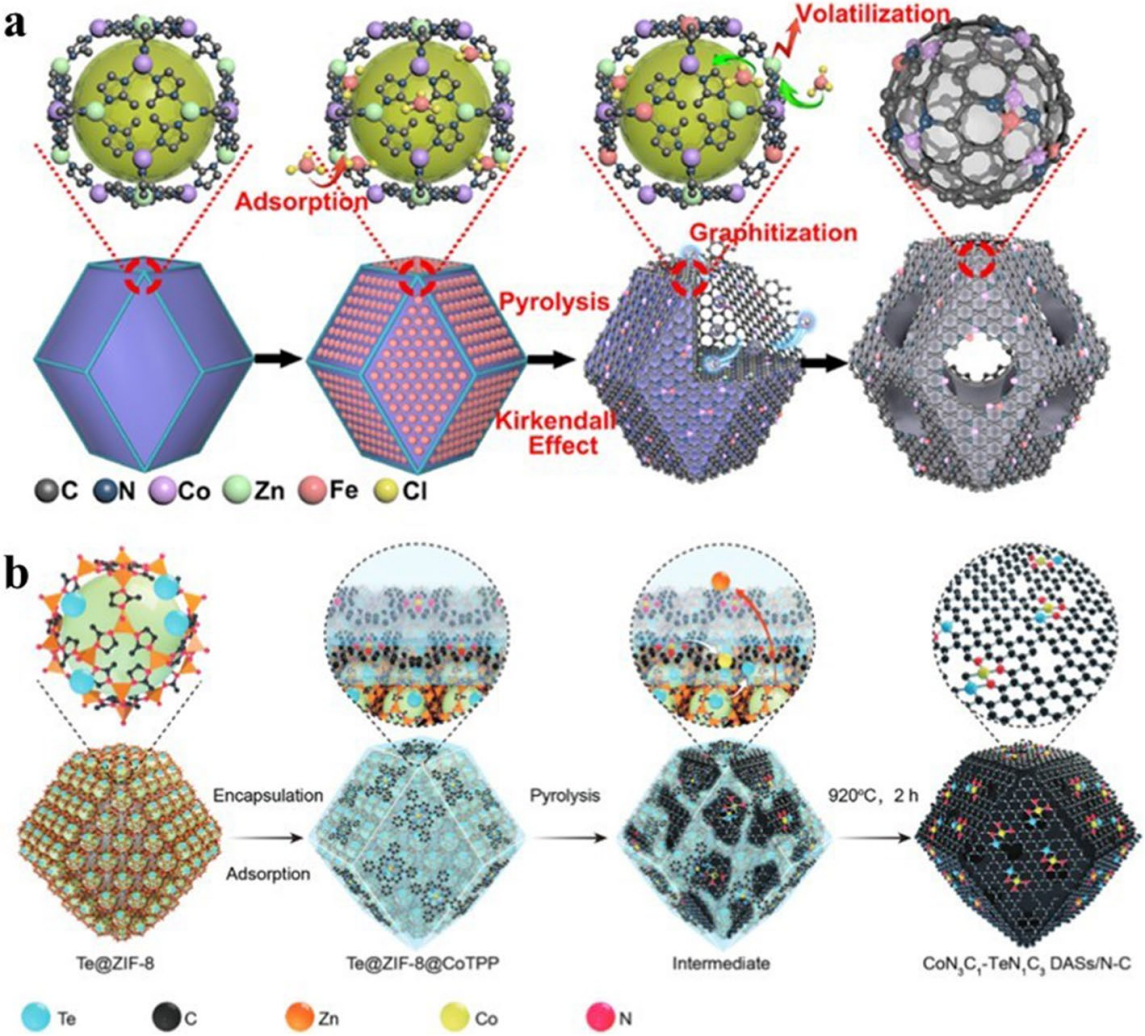
**a** Synthesis pathway of (Fe,Co)/N–C [103]. Copyright 2017 American Chemical Society. **b** Schematic illustration of Co-Te DASs/N–C [104]. Copyright 2022 Wiley–VCH GmbH

In addition to the one-pot strategy, the two-step strategy of constructing dual-metal sites can also achieve the synthesis of DACs. The two-step strategy constructs the desired dual-metal sites by first preparing a precursor containing one of the target metal atoms and then rationally introducing a second metal nearby. Wang et al. prepared (Fe, Co)/N–C DAC through the limited adsorption capacity of bimetallic MOF (BMOF) cavities (Fig. 9a) [103]. To obtain the DAC with dual-metal Fe–Co sites, the Zn/Co BMOF was prepared in the first step. The Fe molecules were successfully introduced into the cavities of the BMOF by simple impregnation and adsorption in the second step. After high-temperature pyrolysis of BMOF, the Zn atoms were able to evaporate and form Fe–Co dual-metal sites successfully in the confined space. Similarly, Wang et al. prepared a DAC with atomically dispersed Co–Te diatomic sites by an encapsulation adsorption pyrolysis strategy (Fig. 9b) [104]. In the first step, the addition of Te powder to the synthetic procedure of ZIF-8 allowed Te atoms to be encapsulated in ZIF-8 cages to yield Te@ZIF-8. In the second step, the tetraphenylporphyrin cobalt (CoTPP) adsorbed on Te@ZIF-8 via *π*–*π* conjugation. During the final high-temperature pyrolysis, the Te atoms diffuse through the ZIF-8 channel into the intermediate carbon layer, while the Co atoms migrate inwards into the intermediate carbon layer leading to the formation of the final Te–Co dual-atom sites.

**Fig. 10:**
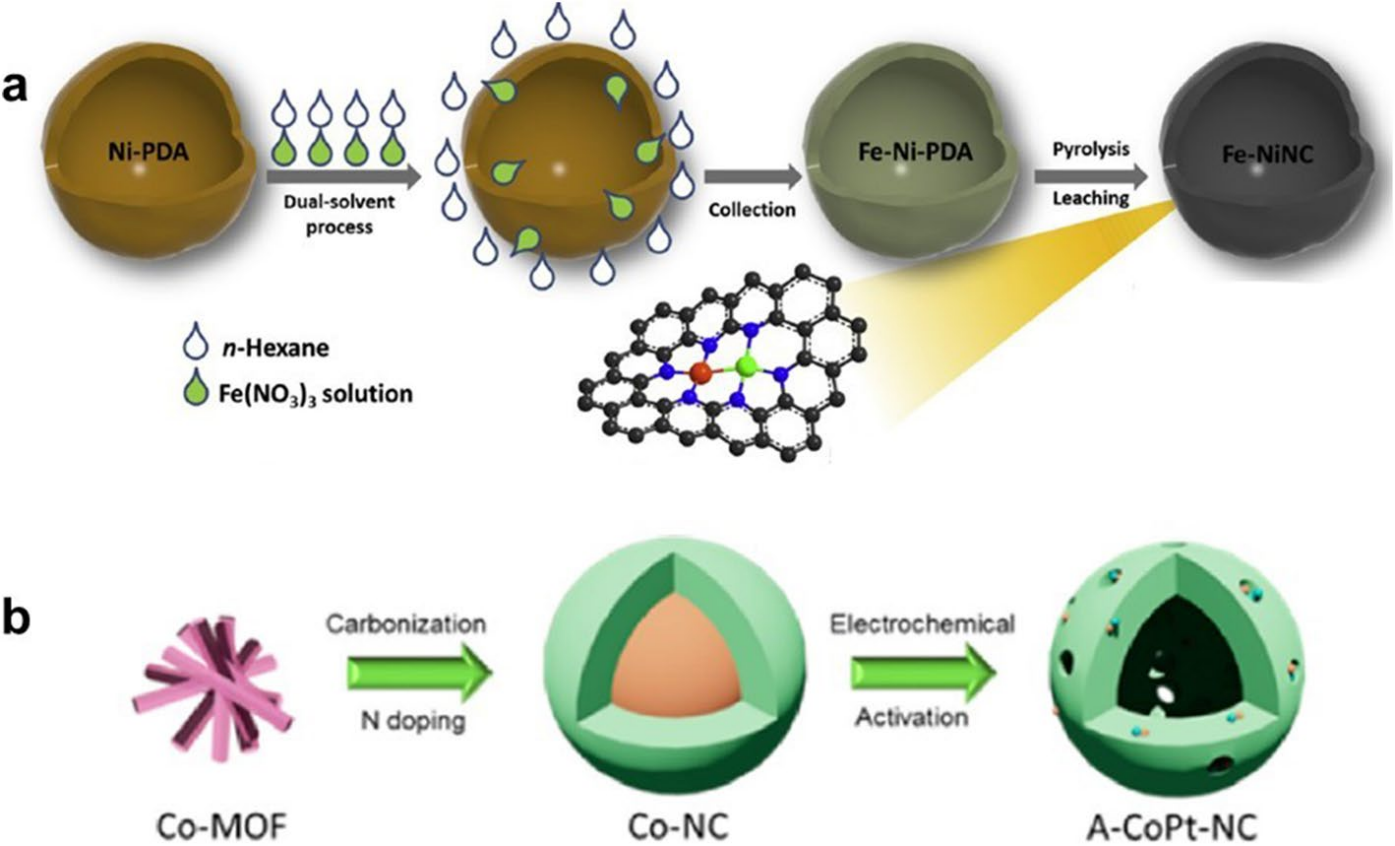
**a S**chematic diagram of the dual-solvent route for Fe–NiNC catalysts [105]. Copyright 2020 Elsevier Ltd. **b** Schematic illustration of A–CoPt–NC [106]. Copyright 2018 American Chemical Society

In Fig. 10a, Zhu et al. synthesized the Fe-NiNC catalyst using a dual-solvent method [105]. In the first step, Ni-doped polydopamine (Ni-PDA) was synthesized as the host to construct Ni sites. In the second step, Fe(NO)_3_ solution is added drop by drop after Ni-PDA has been dispersed in n-hexane. Herein, as hexane and water are immiscible, Fe ions tend to diffuse onto the porous surface of Ni-PDA and reach the Ni site by strong specific adsorption capacity. The Fe–Ni dual-atom sites anchored to the nitrogen-doped carbon are eventually formed by pyrolysis, and the excess metal particles are removed by an acid etching step.

Currently, electrochemical induction strategy has also been adopted to prepare DACs. As shown in Fig. 10b, the DAC that consisted of Co–Pt sites was obtained by a two-step synthesis method [106]. In order to obtain atomically dispersed nitrogen-coordinated Co sites, Co-MOF was carbonized at high temperatures in the first step. To further construct Co–Pt dual-atom sites, an electrochemical activation method was put forward in the second step. In this step, the Co–Pt sites were formed by applying a cyclic potential with the CoNC as the working electrode and the Pt wire as the counter electrode. Although various strategies have been developed, there are always a certain proportion of inevitable single-atom sites in the as-prepared DACs. Controlling the ratio of single to double atom sites precisely has been regarded as a main challenge in DACs synthesis. In addition, how to construct and maintain the conformation of bimetallic pairs during the preparation process also requires further development of more accurate and economical synthesis methods.

## Characterization of DACs

The precise structures of the DACs make it difficult to identify. To demonstrate successful synthesis of DACs, characterize DACs at the atomic scale is urgently needed. The intrinsic activity of the active site is the key parameter that researchers pay attention to. In order to explore the roles of DACs in the target reaction, corresponding structural characterizations are essential. However, the flexible coordination configurations of DACs not only provide more possibilities for the regulation of catalyst activity, but also create more difficulties for the characterization of chemical environment and electronic configuration. The identification of active sites can inspire us to have a better understanding of the reaction mechanism on catalyst surface and provide a reference for further rational design of catalysts. Currently, number of modern sophisticated techniques is exploited to characterize the as-synthesized DACs nanostructures.

### Aberration-Corrected Scanning Transmission Electron Microscopy (AC-STEM)

**Fig. 11:**
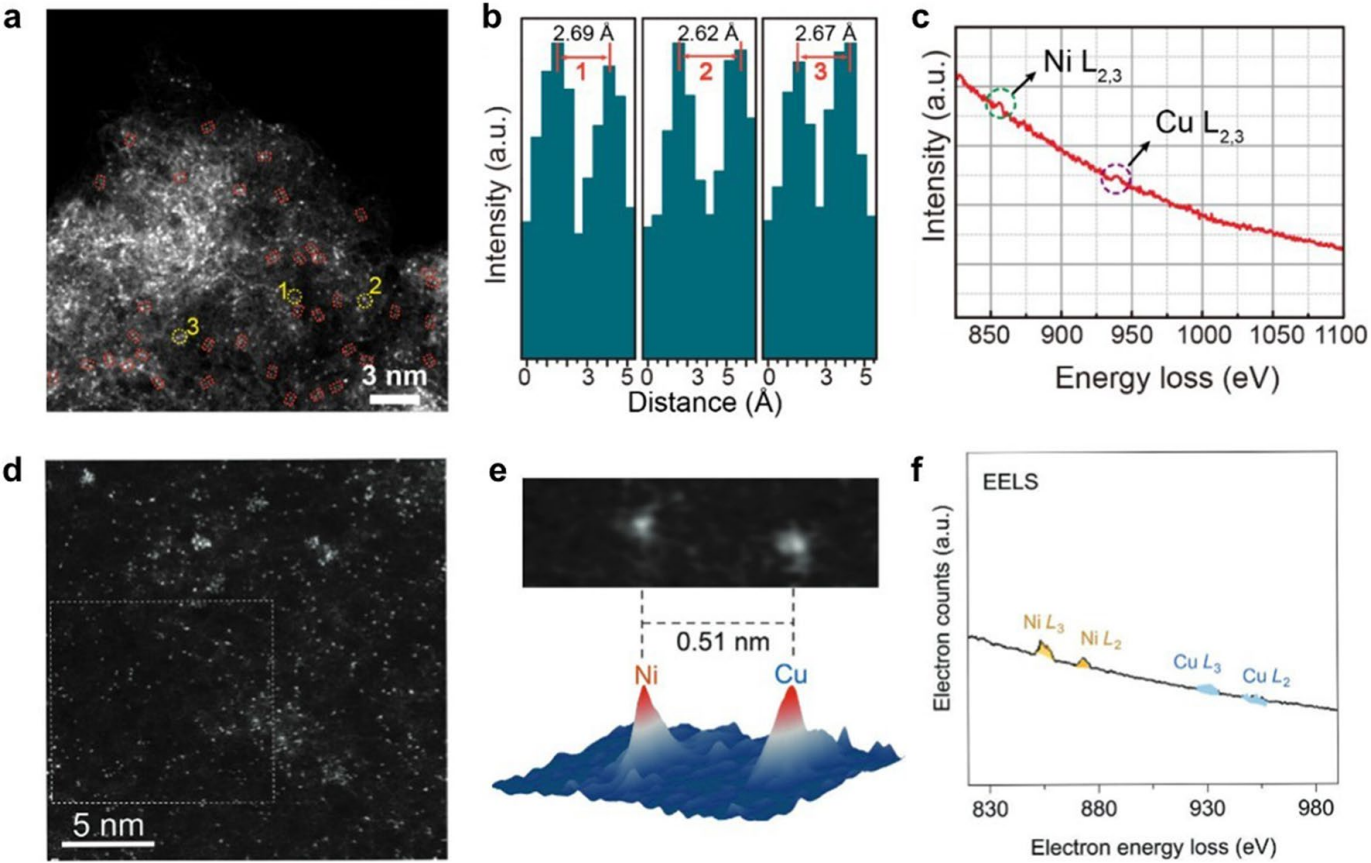
**a** HAADF-STEM image of Cu/Ni-NC. **b** Line-scanning intensity profiles corresponding to the highlights in **a**. **c** EELS diagram of Cu/Ni-NC [47]. Copyright 2023 Wiley–VCH GmbH.** d** HAADF-STEM image of NiCu-NC. **e** NiCu atomic pair and the corresponding 3D intensity profile. **f** EELS diagram of NiCu-NC [108]. Copyright 2023 Wiley–VCH GmbH

Electron microscopic techniques including scanning electron microscopy (SEM), transmission electron microscopy (TEM), and high-resolution transmission electron microscopy (HR-TEM) have been widely used for the morphological characterization of catalysts. However, they are limited by the insufficient resolution and hence not able to accurately characterize DACs. Aberration-corrected scanning transmission electron microscopy (AC-STEM) was developed to address this limitation to achieve sub-angstrom resolution. In particular, aberration-corrected high-angle annular dark field scanning transmission electron microscopy (AC-HAADF-STEM) is able to show metal atoms with larger atomic numbers as prominent highlights, thus allowing visual observation of the presence of diatomic sites [107]. Zhang et al. prepared Ni–Cu binary loaded on a nitrogen-doped carbon substrate [47]. Because the metal and the carbon substrate have a large Z-contrast in atomic number and thus appear as paired bright spots in the AC-HAADF-STEM image, proving the existence of diatomic sites (Fig. 11a), an atomic spacing about 2.6–2.7 Å between metal binary was observed. Such a close distance proves the existence of strong electronic interaction between metal atoms, suggesting the coupling between Cu and Ni atoms (Fig. 11b). The electron energy-loss spectroscopy (EELS) diagram in Fig. 11c reveals the co-existence of Ni and Cu atoms. AC-HAADF-STEM technology makes the diatomic sites “visible”; thus, the classification of DACs can be identified to some extent. A NiCu DAC loaded on a nitrogen-doped carbon substrate was prepared by Yao et al. [108]. They demonstrated that the Ni and Cu atoms have the best catalytic performance when they are in close proximity to each other up to 5.3 Å by theoretical simulations. In Fig. 11d, the bright dots in the AC-HAADF-STEM image prove the presence of the diatomic sites. The atomic spacing of 0.51 nm proves that the metal sites in the DAC are close to each other but not next to each other (Fig. 11e). Therefore, the NiCu DAC can be well distinguished from other configurations of DACs. Similarly, the coexistence of Ni and Cu sites can be confirmed by the EELS diagram (Fig. 11f). Although AC-HAADF-STEM has an irreplaceable advantage for the observation of metal sites, they are hardly useful for the characterization of the chemical state of metal sites and nearby coordination atoms. To investigate the structural information of DACs in more detail, the combination of additional characterization tools is needed.

### X-ray Techniques

**Fig. 12:**
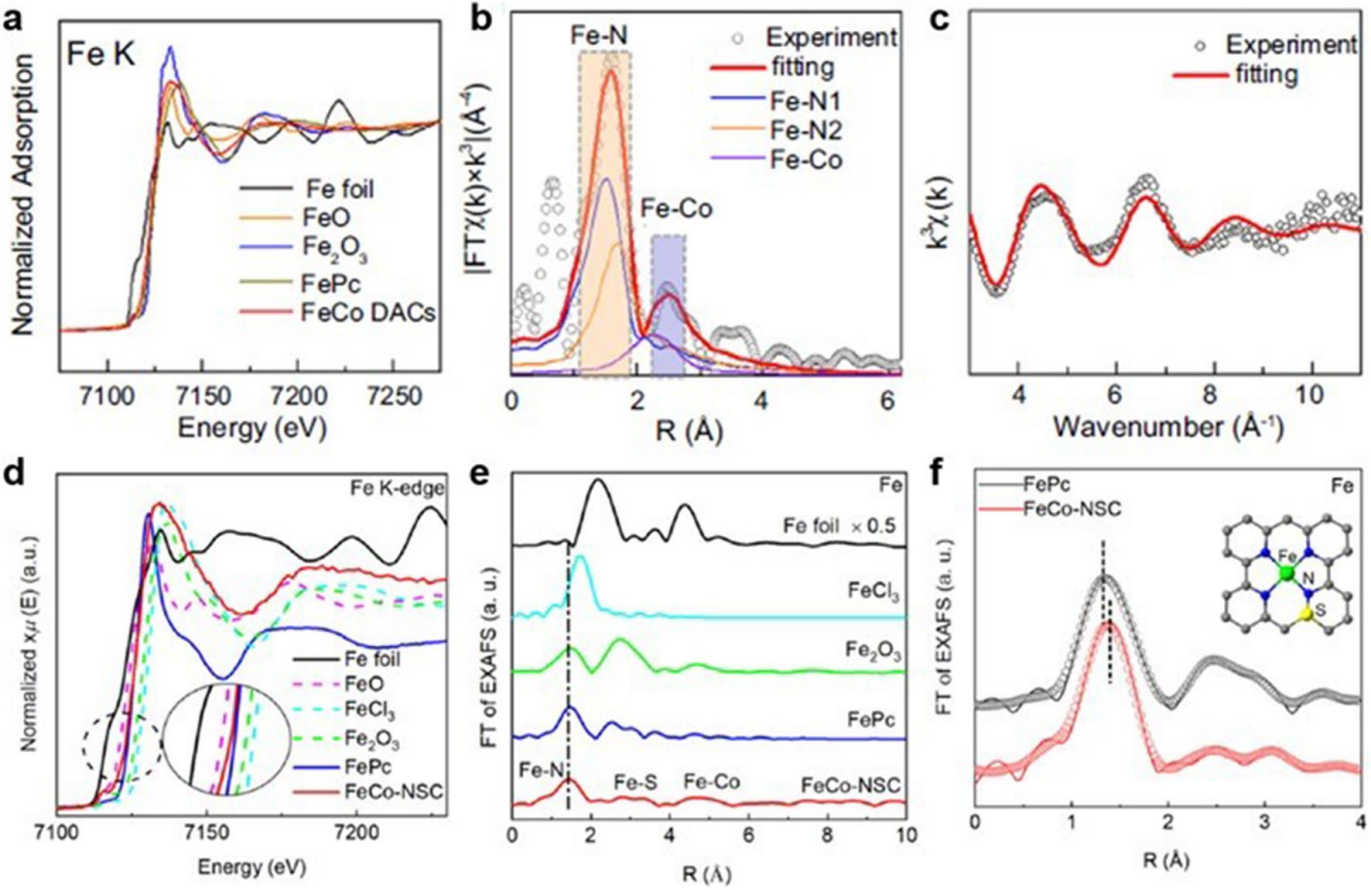
**a** Fe K-edge XANES spectra. **b** EXAFS fitting curve of Fe in Fe–Co DACs spectra. **c** k-space experimental EXAFS spectra and fitting curves of Fe in Fe–Co DACs [46]. Copyright 2023 Springer Nature. **d** Fe K-edge XANES spectra. **e** FT-EXAFS spectra. **f** Corresponding fitting in R space of FeCo-NSC and FePc at Fe K-edge [43]. Copyright 2022 Elsevier Ltd

X-ray characterization techniques have been widely used for structural studies of DACs. The common X-ray characterization mainly includes X-ray diffraction (XRD), X-ray photoelectron spectroscopy (XPS), and X-ray absorption spectroscopy (XAS). XRD can be used to determine whether metal atoms form relevant nanoparticles, but it cannot directly identify the presence of atomic-level dispersed sites. XPS can reflect the valence information of atoms and coordination structures, but the results obtained are not accurate and limited by the poor signal due to low metal loading. Therefore, to more accurately characterize the active sites of DACs, XAS has been proposed and achieved favourable effect. XAS contains extended X-ray absorption near-edge structure (XANES) and extended X-ray absorption fine structure (EXAFS) [109]. XANES provides a good representation of the electronic orbits and valence states of the atoms in DACs, while EXAFS provides information on the type of coordination atoms, coordination number, bond length, and even the structure of the adjacent coordination shell after Fourier transformation. XANES and EXAFS play important roles in revealing the microstructure of DACs and assisting in establishing the relationship between activity and structure. The characterization of FeCo DACs is taken as an example to illustrate the application of XAS to the local structure measurement of the active sites in DACs [46]. Firstly, the position of the pre-edge in the XANES spectra can visually reflect the valence information of the metal sites. In Fig. 12a, the pre-edge of FeCo DACs in Fe K-edge XANES spectra is located between standard Fe foil and Fe_2_O_3_, revealing that the valence state of Fe is between 0 and + 3. To obtain fine structure information, the Fourier transform (FT) *k*^3^-weighted χ(*k*)-function of Fe K-edge EXAFS in R space is further proposed (Fig. 12b). The R-space spectra of FeCo DACs were fitted to reveal three different scattering paths corresponding to Fe–N_1_, Fe–N_2_, and Fe–Co. The existence of Fe–Co bonds is attributed to the strong interaction between Fe–Co, proving that Fe and Co atoms are close together in the diatomic sites. The fitting results show that the coordination numbers of Fe–N and Fe–Co are 3.1 and 0.7, respectively, revealing the fine structural information of Fe–Co DACs. In addition, the fitting result of the K space spectrum for FeCo DACs also has good agreement with the experiment spectrum, further identifying the reliability of the structure (Fig. 12c). Unlike the above, Wu et al. prepared and characterized another configuration of Fe–Co DAC in which Fe and Co atoms are close to each other but not adjacent proximity [43]. The absorption edge in Fe K-edge spectra suggested the average valence state of Fe species in the as-prepared FeCo-NSC was between + 2 and + 3 (Fig. 12d). Different from the former work, FeCo-NSC in the Fourier transform (FT) *k*^3^-weighted EXAFS spectrum showed only one major peak at 1.47 Å, revealing the presence of Fe–N bonds (Fig. 12e). The absence of Fe–Co peak proved that the Fe and Co atoms were separated by a certain distance. Similarly, the results of R space fitting further investigated the fine structure of Fe–Co-NSC, where the coordination number of Fe–N was shown to be 4 and the presence of a sulphur atom in the second coordination shell layer (Fig. 12f). As a widely used structural detection technology, XAS has shown great advantages in the characterization of fine structures of DACs. However, XAS is facing an obvious drawback of providing only average structural information. To obtain more precise structural information of DACs, more evidence should be provided further.

### In Situ Characterization

**Fig. 13:**
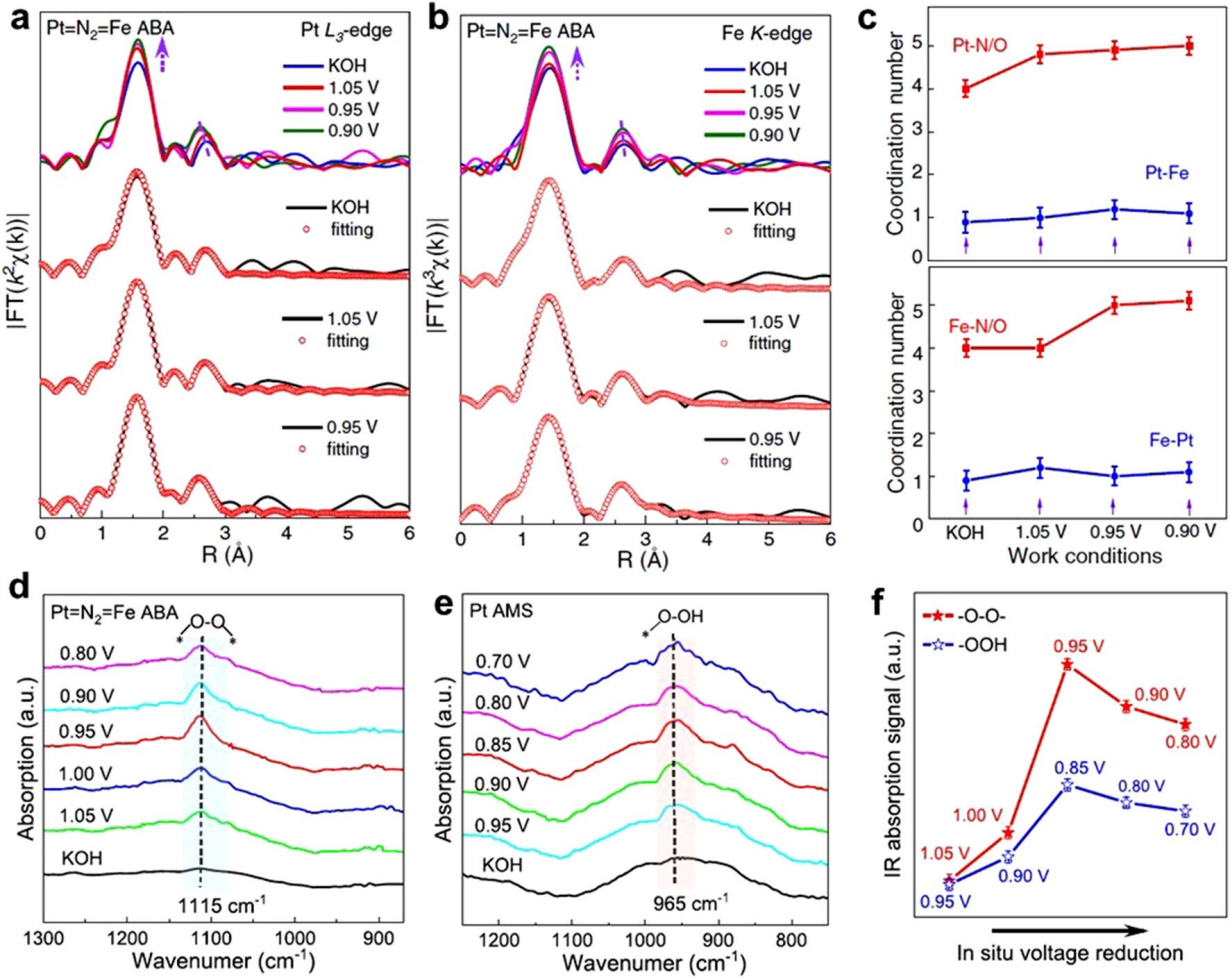
**a** Pt L_3_-edge and **b** Fe K-edge FT-EXAFS spectra and the corresponding fitting curves under different potentials. **c** The fitting results of the coordination number. In situ SR-FTIR characterizations for **d** Pt = N_2_ = Fe ABA and **e** Pt AMS. **f** FTIR absorption stretching for Pt = N_2_ = Fe ABA and Pt AMS [40]. Copyright 2022 Springer Nature

The role of DACs in catalytic reactions and the study of their reaction mechanisms are the focus of current research. However, the complex electron transfer processes and uncertain reaction intermediates in various reactions have hindered further studies on DACs. Therefore, there is an urgent need to develop in situ techniques for observing the evolution of active sites of DACs under real operating conditions. Zhou et al. developed a DAC with nitrogen-bridged Pt = N_2_ = Fe sites for efficient four-electron ORR [40]. To understand the origin of the high kinetics and selectivity of Pt = N_2_ = Fe sites at the atomic level, they employed in situ synchrotron radiation techniques to study the structural evolution of Pt = N_2_ = Fe sites at different operating voltages. The FT-EXAFS spectra of Pt L_3_-edge showed a significant enhancement of the main peak signal intensity at 1.55 Å under operating voltage compared to the ex*-*situ condition (Fig. 13a). The similar tendency was also seen in the FT-EXAFS spectra of the Fe K-edge, proving that both Pt and Fe atoms were engaged in the reaction during the ORR process (Fig. 13b). The corresponding fitting results showed that the coordination numbers of Pt–N and Fe–N are both 4. Additional Pt–O coordination and Fe–O coordination appear of 1.05 and 0.95 V, respectively, demonstrating the adsorption configuration of the oxygen intermediate was Pt–O–O–Fe (Fig. 13c). The Pt–O–O–Fe configuration facilitates the breakage of O–O bond which is considered to be an important factor for achieving efficient four-electron ORR. Furthermore, in situ synchrotron radiation Fourier transform infrared spectroscopy (SR-FTIR) was also used for the detection of reaction intermediates. The detection of the intermediate O–O likewise proves the formation of the Pt–O–O–Fe configuration (Fig. 13d). In contrast, the ORR intermediate signal detected at the Pt site of corresponding SAC was *OOH, revealing a different ORR reaction pathway. On Pt = N_2_ = Fe sites (Fig. 13e), there was an increasing intensity -O–O- signal after an operating voltage of 1.05 V (Fig. 13f). The –O–O- signal on Pt = N_2_ = Fe was much higher than the *OOH signal on the corresponding single-atom site. This well explained the origin of the more excellent four-electron ORR catalytic performance of DACs compared to SACs. Current in situ techniques have only elucidated the reaction mechanism of part of DACs, while more DACs remain to be systematically investigated. In addition, few in situ studies have been reported for working conditions in real equipment. In situ characterization techniques still need more extensive and in-depth research.

## Application of DACs in Electrocatalysis

DACs possess same advantages of high unsaturated coordination and maximal atomic utilization as SACs [110, 111]. More than that, DACs also show higher tunability, wider availability of denser active sites than SACs, thus providing more opportunities to diverse reactions. The synergistic effects between two metal sites in DACs can effectively regulate the catalytic activity and stability, leading to a wide range of applications, including oxygen reduction reaction, carbon dioxide reduction reaction, hydrogen evolution reaction, nitrogen reduction reaction, etc. [30, 112–115].

### Oxygen Reduction Reaction (ORR)

Oxygen reduction reaction plays a key role in energy conversion equipment such as fuel cells and metal-air batteries [116–118]. The sluggish kinetic of ORR requires precious metal platinum as catalyst to accelerate the reaction [119, 120]. The high cost and low availability of Pt hinder the application of fuel cells [3, 121]. Therefore, there is an urgent need to develop nonprecious metal ORR catalysts with high activity and stability [122]. Atomically dispersed catalysts show great potential in ORR and are considered as promising candidates for noble metal catalysts [123]. Wang et al. applied DACs to acidic ORR for the first time and prepared (Fe,Co)/N–C with ultra-high catalytic activity and stability [103]. DFT calculations demonstrated that the diatomic sites have unique advantages in O–O activation. Subsequently, DACs have received increasing attention and have been recognized as promising candidates for efficient ORR catalysts.

The homonuclear DACs, such as Co_2_ [124] and Fe_2_ [93, 112], have shown considerable ORR activity. The quality activity of the Co_2_N_*x*_C_*y*_ DAC prepared by Xiao et al. is about 12 times than CoN_4_ SAC. The half-wave potential of Co_2_N_*x*_C_*y*_ in acidic electrolyte was 0.79 V, close to the commercial Pt/C catalyst [124]. The Fe_2_–N–C synthesized by Ye et al. also presented promising activity of ORR in acidic medium [93]. The half-wave potential was only 20 mV lower than that of commercial Pt/C. Moreover, the planar Fe_2_N_6_ structure synthesized by Zhang et al. possessed a mass activity 700% higher than that of the separated FeN_4_ structure [112]. The unique DAC structure demonstrated a half-wave potential of 0.84 V under acidic conditions, which was comparable to commercial Pt/C.

**Fig. 14:**
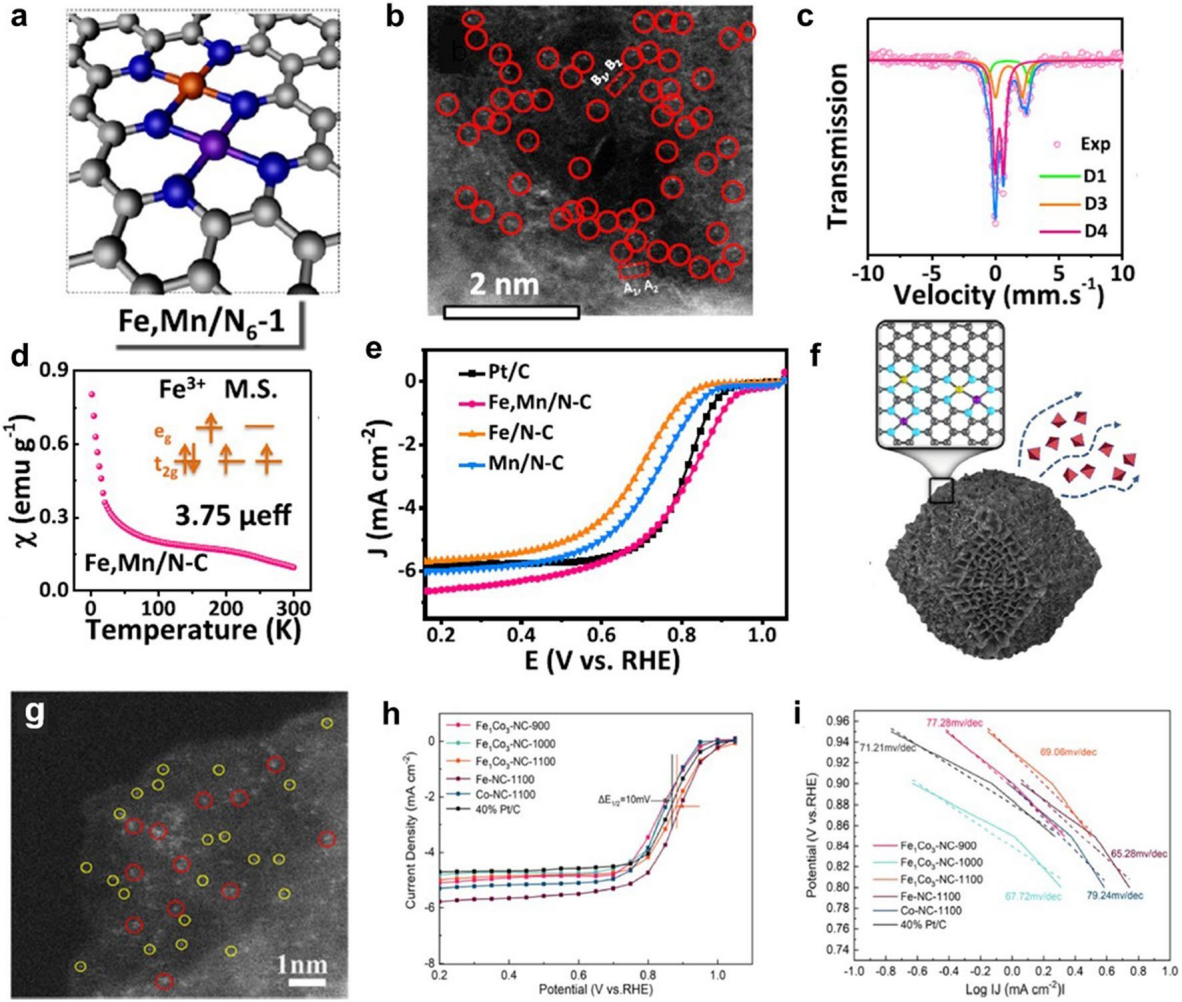
**a** The optimized structure model, **b** aberration-corrected HAADF-STEM image, **c** ^57^Fe Mossbauer spectrum. **d** magnetic susceptibility of Fe,Mn/N–C. **e** LSV curves of Fe,Mn/N–C, Fe/N–C, Mn/N–C and Pt/C catalyst in 0.1 M HClO_4_ solution [132]. Copyright 2021 Springer Nature. **f** Structure model, **g** HAADF-STEM image of FeCo-NC. **h** SCV measurements, **i** Tafel plots of the as-prepared catalysts 0.1 M KOH solution [128]. Copyright 2022 American Chemical Society

In addition to homonuclear DACs, heteronuclear DACs have been widely studied due to their diversity of active site combinations [125]. The active sites of metal pairs like FeCo [110, 126–128], FeZn [129], ZnCo [74, 130, 131], FeMn [132, 133], FeNi [48, 105, 134], FeCu [135], CoPt [106], CoNi [136], AuCo [84] exhibit superior ORR activity. Among them, Yang et al. successfully prepared Fe,Mn/N–C electrocatalyst by the prepolymerization and pyrolysis processes (Fig. 14a) [132]. The iron phthalocyanine (FePc) and manganese nitrate (Mn(NO_3_)_2_) were used as metal sources. The aberration-corrected HAADF-STEM image in Fig. 14b demonstrated that metal atoms were atomically dispersed. This work supplies a robust strategy for enhancing the catalytic activity of ORR by regulating Fe-spin state. The ^57^Fe Mossbauer spectrum exhibited that Fe^III^ with the medium-spin structure was dominant after constructed Fe–Mn dual metal pair sites (Fig. 14c), which was responsible for a better catalytic performance. To further clarify the electron spin configuration, the zero-field cooling (ZFC) temperature-dependent magnetic susceptibility was conducted (Fig. 14d). The results showed that the low-spin state of adjacent Mn^III^ induced Fe^III^ to possess a reasonable e_g_ filling. This optimization endows Fe,Mn/N–C with superior ORR activity with a half-wave potential of 0.804 V in 0.1 M HClO_4_ (Fig. 14e). Recently, He et al. synthesized an atomically dispersed Fe–Co DAC by introducing Fe^3+^ and Co^2+^ ions into the ZIF-8 (Fig. 14f) [128]. Atomically dispersed Fe–Co sites embedded in the carbon framework were obtained by adjusting the proportion of metal ions and subsequent pyrolysis procedure (Fig. 14g). The Fe_1_Co_3_-NC-1100 showed a better activity in half-wave potential of 0.877 V than commercial 40% Pt/C (Fig. 14h). Furthermore, the Fe_1_Co_3_-NC-1100 exhibited lowest Tafel slopes of 69.06 mV dec^−1^, uncovering an excellent kinetics process (Fig. 14i).

Although DACs have shown high ORR activity in the rotating disk electrode (RED), their performance in membrane electrode assembly (MEA) applications has yet to be improved. How to improve the activity of DACs in MEA will be one of the main research directions of ORR electrocatalysts in the future. The application potential of DACs has often been evaluated in recent years through energy-related devices such as Zn-air batteries and fuel cells. Notably, DACs are able to maintain remarkably high catalytic activity in alkaline electrolytes, even much better than commercial Pt/C 20% catalysts. As a result, DACs as cathodes can surpass the performance of commercial Pt/C 20% in Zn-air batteries and continue to break through in peak power density (Table 1). However, DACs often have difficulty exceeding the peak power density output of commercial Pt/C in more complex alkaline exchange membrane fuel cells (AEMFCs). In addition, DACs performed less well in acidic media than in alkaline media and suffered from the same lack of durability as SACs. Therefore, exploring the reaction mechanism and deactivation mechanism of DACs for the actual working conditions of membrane electrodes will be the main task for DACs.

**Table 1:** The activity of DACs for ORR and their performance in different applications

Catalyst	E1/2 (V vs. RHE)	Electrolyte	Application	Peak power density (mW cm^−2^)	Refs
Fe–Se/NC	0.925	0.1 M KOH	Zn-air batteries	135	[137]
FeCo-NSC	0.86	0.1 M KOH	Zn-air batteries	152.8	[43]
FeCo-NC	0.877	0.1 M KOH	Zn-air batteries	372	[128]
CoFe-NG	0.952	0.1 M KOH	Zn-air batteries	230	[138]
Fe,Zn–N–C	0.867	0.1 M KOH	Zn-air batteries	138	[139]
Fe2DAC	0.898	0.1 M KOH	Zn-air batteries	325.8	[140]
D-FeCo-DAs-N–C	0.927	0.1 M KOH	Zn-air batteries	259	[141]
FeCo/DA@NC	0.84	0.1 M KOH	Zn-air batteries	110.3	[142]
Cu–Co/NC	0.92	0.1 M KOH	Zn-air batteries	295.9	[143]
Zn/Fe-NC	0.875	0.1 M KOH	Zn-air batteries	186.2	[144]
Fe–Mn–N–C	0.93	0.1 M KOH	AEMFCs	1321	[36]
FeCu-NC	0.882	0.1 M KOH	AEMFCs	910	[145]
FeCo-MHs	0.95	0.1 M KOH	AEMFCs	604.9	[146]
Fe–Mn–N–C	0.79	0.1 M HClO_4_	PEMFCs*	1048	[36]
Fe, Cu DAs-NC	0.80	0.5 M H_2_SO_4_	PEMFCs	875	[147]
Fe&Pd–C/N	0.808	0.5 m H_2_SO_4_	PEMFCs	362	[148]
Cu–Co/NC	0.85	0.5 m H_2_SO_4_	PEMFCs	963	[143]
FeMo–N–C	0.84	0.1 M HClO_4_	PEMFCs	460	[149]
FeCe-SAD/HPNC	0.81	0.1 M HClO_4_	PEMFCs	771	[150]
(Au–Co) DP-NPAs	0.82	0.1 M HClO_4_	PEMFCs	490	[84]

*PEMFC represents proton exchange membrane fuel cell

### Carbon Dioxide Reduction Reaction (CO_2_RR)

Electrocatalytic reduction reaction of CO_2_ is a potential way to solve the current energy crisis and carbon emission problems [151, 152]. Through CO_2_RR, CO_2_ can be reduced to various value-added products such as carbon monoxide (CO), formic acid (HCOOH), methanol (CH_3_OH), ethylene (CH_2_CH_2_), ethanol (C_2_H_5_OH) and even longer carbon chains [153]. However, the HER as a competitive reaction is easy to occur during electrolysis, leading to low Faraday efficiency [154–156]. The stable C=O bond and HER competition in CO_2_RR require catalysts with high-activity and excellent selectivity [157, 158]. For the electrocatalysis of CO_2_RR, low over potentials are difficult to achieve due to the scaling relationships of the adsorbents (in particular *COOH, *CO and *CHO) [159]. Due to the unique structural and electronic properties, the construction of DACs is expected to be an effective way to optimize the adsorption strength of intermediates. DACs are expected to provide assistance in breaking scaling relationships of the adsorbents and simultaneously reducing the absorption of H to suppressing the side reaction.

**Fig. 15:**
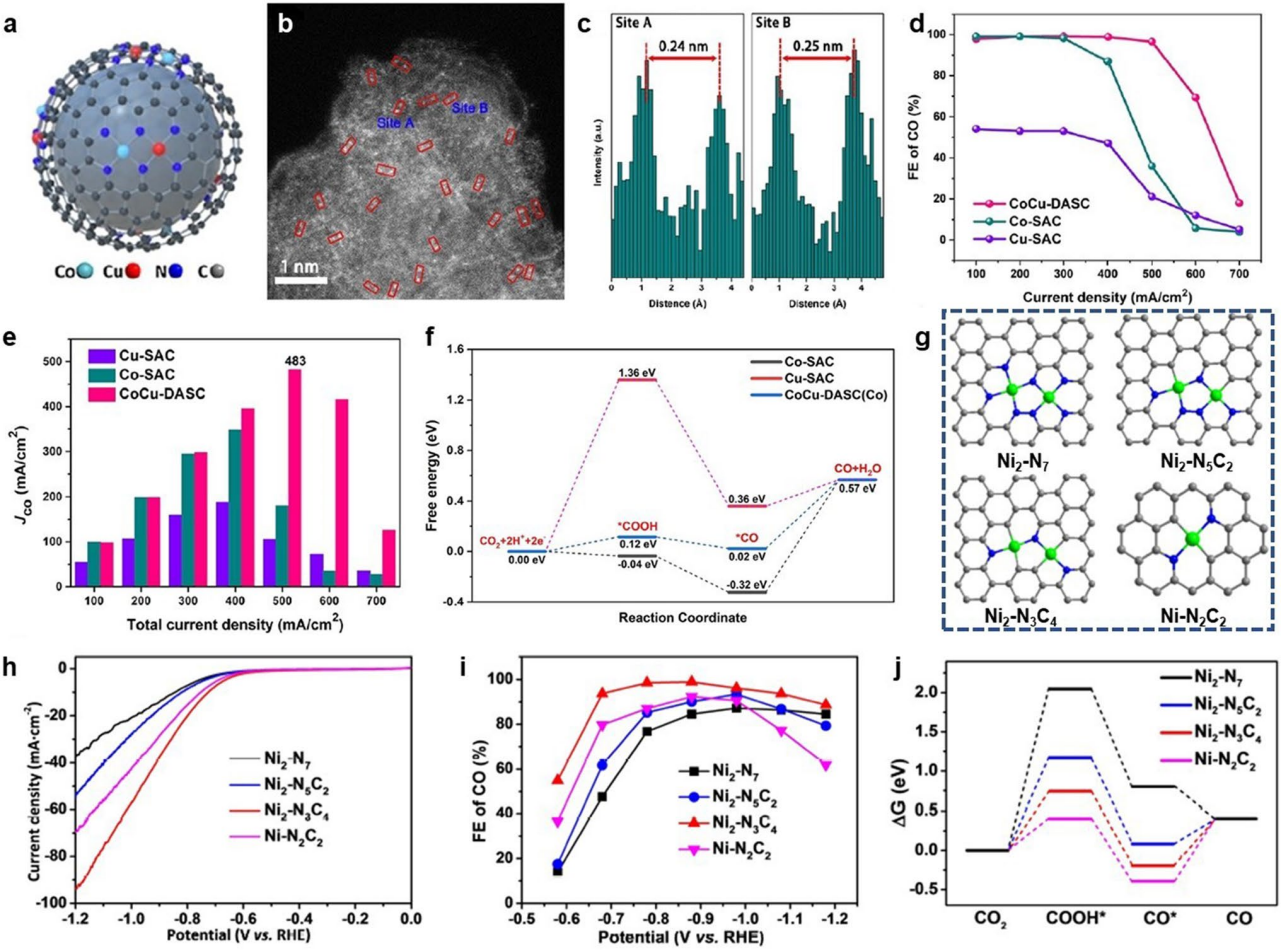
**a** Illustration and **b** HAADF-STEM image of CoCu-DASC. **c** Line-scanning intensity profiles obtained from the site A and B in panel **b. d** Faradaic efficiencies for electrochemical reduction of CO_2_ to CO, **e** CO partial current density in flow cell and **f** free energy profiles of CoCu-DASC and corresponding SACs [161]. Copyright 2022 Wiley–VCH GmbH **g** Illustration, **h** LSV curves, **i** Faraday efficiencies for electrochemical reduction of CO_2_ to CO and **j** free energy profiles of Ni_2_ DACs and Ni SAC [162]. Copyright 2022 Wiley–VCH GmbH

**Fig. 16:**
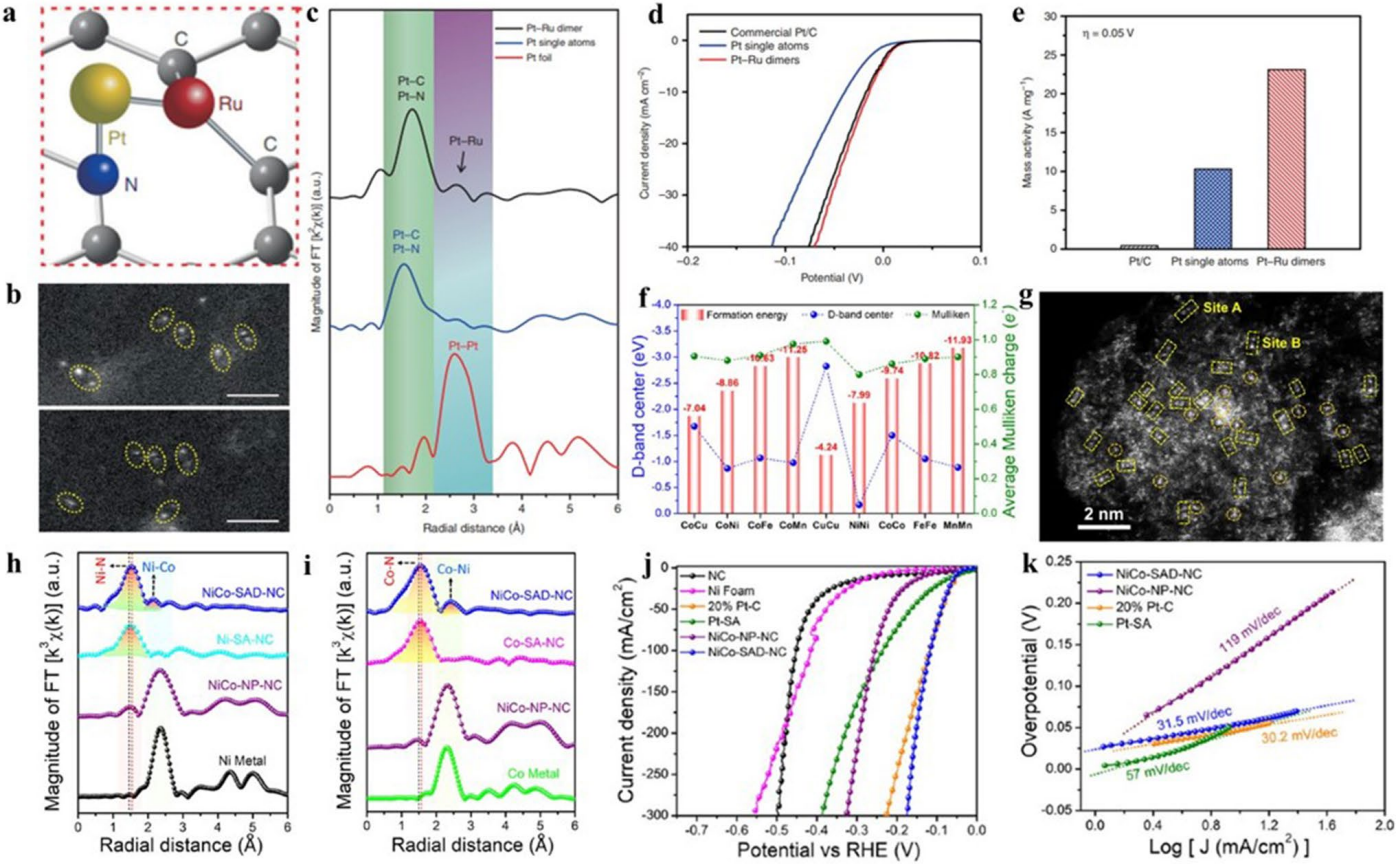
**a** Schematic diagram of Pt–Ru dimers on NCNT. **b** HAADF-STEM images of Pt–Ru dimers. **c** K^2^-weighted magnitude of Fourier transform spectra of Pt–Ru dimers and Pt single atoms. **d** HER polarization curves and **e** normalized mass activity of Pt–Ru dimers and Pt single atoms at 0.05 V [171]. Copyright 2019 Springer Nature **f** DFT calculations of formation energies and d-band centre for bimetallic single-atom dimers. **g** HAADF-STEM image of NiCo-SAD-NC. **h** Ni K-edge and **i** Co K-edge FT-EXAFS spectra. **j** LSV curves and **k** corresponding Tafel plots [172]. Copyright 2021 Springer Nature

CO_2_RR has a variety of possible reaction pathways, resulting in different intermediates and products. Zhang et al. prepared a supported Pd_2_ DAC by anion replacement deposition–precipitation strategy [160]. The binuclear Pd(II) complexes were pre-synthesized to construct diatomic Pd_2_ sites. According to the electrochemical activity test, the Pd_2_ DAC exhibited the highest Faraday efficiency even reaching 98.2% at − 0.85 V, solving the problem of insufficient selectivity on Pd-based catalysts. The stability measurement was conducted at − 0.8 V. The Faraday efficiency and *j*_total_ current density showed an insignificant change after 12 h. Yi et al. attempted to construct a Co–Cu DAC (denoted as CoCu-DASC) to solve the current problem that related SACs cannot achieve industrial-level current density and high selectivity in CO_2_RR at the same time (Fig. 15a) [161]. CoCu-DASC was obtained by pyrolysis of conductive carbon black with a simple mixture of metal salts and urea. HAADF-STEM image of CoCu-DASC demonstrated that the metal atoms were uniformly dispersed at the atomic level (Fig. 15b) and form atomic pairs with a spacing of 2.4–2.5 nm (Fig. 15c). Electrochemical catalytic reduction of CO_2_ to CO tested in a liquid flow cell system showed that CoCu-DASC was capable of achieving Faraday efficiency above 90% over a wide range of current density (Fig. 15d). Current density in the Co partial can be as high as 483 mA cm^−2^ at a total current of 500 mA cm^−2^, well above the industrial-level requirements (Fig. 15e). Theoretical calculations demonstrated that the synergistic interaction between Co and Cu sites can effectively reduce the activation energy of *COOH and promote *CO desorption (Fig. 15f), thus realizing efficient CO_2_RR. Ouyang et al. calculated 21 kinds of heteronuclear transition metal atomic pairs embedded in a single layer of C_2_N [159]. Among them, CuCr/C_2_N and CuMn/C_2_N showed low limiting potentials of − 0.37 and − 0.32 V. It proved that CO_2_ can be effectively reduced to CH_4_, which provides a reference for future experimental design. The effect of the coordination microenvironments of Ni_2_ DACs on CO_2_RR was first studied by Gong et al. [162]. A dinuclear nickel complex [Ni_2_(L^1^)_2_(L^2^_)2_(H_2_O)_2_]·2H_2_O (L^1^ = adenine; H_2_L^2^ = malonic acid) was used to prepare Ni_2_ DACs. The modulation of the coordination microenvironment was achieved by modifying the pyrolysis temperature to produce Ni_2_-N_7_, Ni_2_–N_5_C_2_ and Ni_2_–N_3_C_4_ at 700, 800 and 900 °C, respectively (Fig. 15g). As shown in Fig. 15h, Ni_2_–C_3_N_4_ provided best onset potential and highest current density. Further measurements found Ni_2_–C_3_N_4_ to have excellent performance in the electrocatalytic reduction of CO_2_ to CO, demonstrating a Faraday efficiency up to 98.9% (Fig. 15i). DFT calculations revealed that Ni_2_C_3_N_4_ had the optimal binding energy for *COOH and *Co intermediates (Fig. 16j), demonstrating that the coordination microenvironment has an essential effect on the catalytic activity of DACs.

### Hydrogen Evolution Reaction (HER)

Hydrogen is considered to be one of the most desirable clean energy sources. As a highly efficient and pollution-free energy, hydrogen produces only water during combustion [163]. Electrocatalytic water splitting is a significant pathway to produce hydrogen which shows zero emission of harmful greenhouse gases [164]. However, the exaggerated overpotential of HER has become one of the most troublesome barrier for the popularize of the electrocatalytic water splitting [165]. Although the conventional platinum-based catalysts have exhibited favourable performance in HER, their scarcity and high costs have severely affected commercialization. Therefore, it is necessary to develop a credible substitute for platinum-based catalysts with high efficiency towards HER [166–168]. Carbon-based SACs have shown great potential in HER because of their good conductivity and adjustable electronic structure [169]. In theory, the Heyrovsky reaction (H* + H_2_O + e^−^ → H_2_ + OH^−^) and Tafel reaction (H* + H* → H_2_) in HER are more likely to occur at two adjacent sites [170]. Therefore, compared with SACs, DACs are considered to be more promising catalysts for HER.

By atomic layer deposition (ALD) technology, Zhang et al. successfully prepared Pt–Ru DAC for high-efficiency HER (Fig. 16a) [171]. As shown in HAADF-STEM images, the bright dots in pair revealed the formation of dual metal dimers (Fig. 16b). The electronic environment of Pt–Ru DAC was further investigated by XAS measurements. The Fourier transforms (FT) spectra of the Pt EXAFS clearly demonstrated the existence of Pt–Ru bonds (Fig. 16c). The as-prepared Pt–Ru DAC exhibited excellent HER catalytic performance, with the mass activity up to 23.1 A mg^−1^ at 0.05 V (Fig. 16d, e). Kumar et al. high-throughput screened a series of bimetallic single-atom dimer (SAD) to obtain an optimal structure for HER [172]. Among them, NiCo dimer showed low *d*-band centre and formation energy (Fig. 16f). Therefore, NiCo SAD was further investigated and prepared. HAADF-STEM image suggested high density of metal dimers was successfully constructed (Fig. 16g). The local structure environment of Ni and Co was characterized by EXAFS, which demonstrated the formation of Ni–Co dimers (Fig. 16h, i). The LSV measurement was taken to evaluate the activity of NiCo SAD in HER (Fig. 16j). The NiCo SAD showed superior activity for HER with low overpotential of 54.7 mV at 10 mA cm^−2^ and low Tafel slope of 31.5 mV dec^−1^ (Fig. 16k).

### Nitrogen Reduction Reaction (NRR)

Ammonia is one of the important inorganic chemical products, which is mainly used in the synthesis of fertilizer, chemical, and pharmaceutical raw materials. The large scale of NH_3_ production in industry is still dominated by the traditional Haber–Bosch (H–B) process under high temperature and high pressure [173]. The process is not only energy-intensive, but also produces high CO_2_ emissions [174, 175]. Therefore, in recent years, researchers have paid more attention to the electrochemical nitrogen reduction reaction (NRR), which catalyses the production of NH_3_ by atmospheric nitrogen under environmental conditions [176, 177]. However, it is difficult for nitrogen to be activated effectively due to the high nitrogen triple bond energy of N_2_. In addition, the Faraday efficiency of NRR is greatly limited by HER as a side reaction [178, 179]. Electrocatalysts for NRR with high activity and selectivity are urgently needed. Rational design of SACs has been proven to be a reliable way to inhibit HER, thus improving Faraday efficiency. However, it is very challenging for SACs to achieve a satisfactory performance in NRR. It could be ascribed to the sluggish kinetics of the first and last proton-coupled electron transfer on the unitary single-atom site [114]. Besides, the NRR involves multiple reaction intermediates, resulting in scaling relationships that hinder further improvements in yield and Faraday efficiency. To solve the problems above, the researchers further explored the possibility of using DACs in NRR [114, 180–182].

**Fig. 17:**
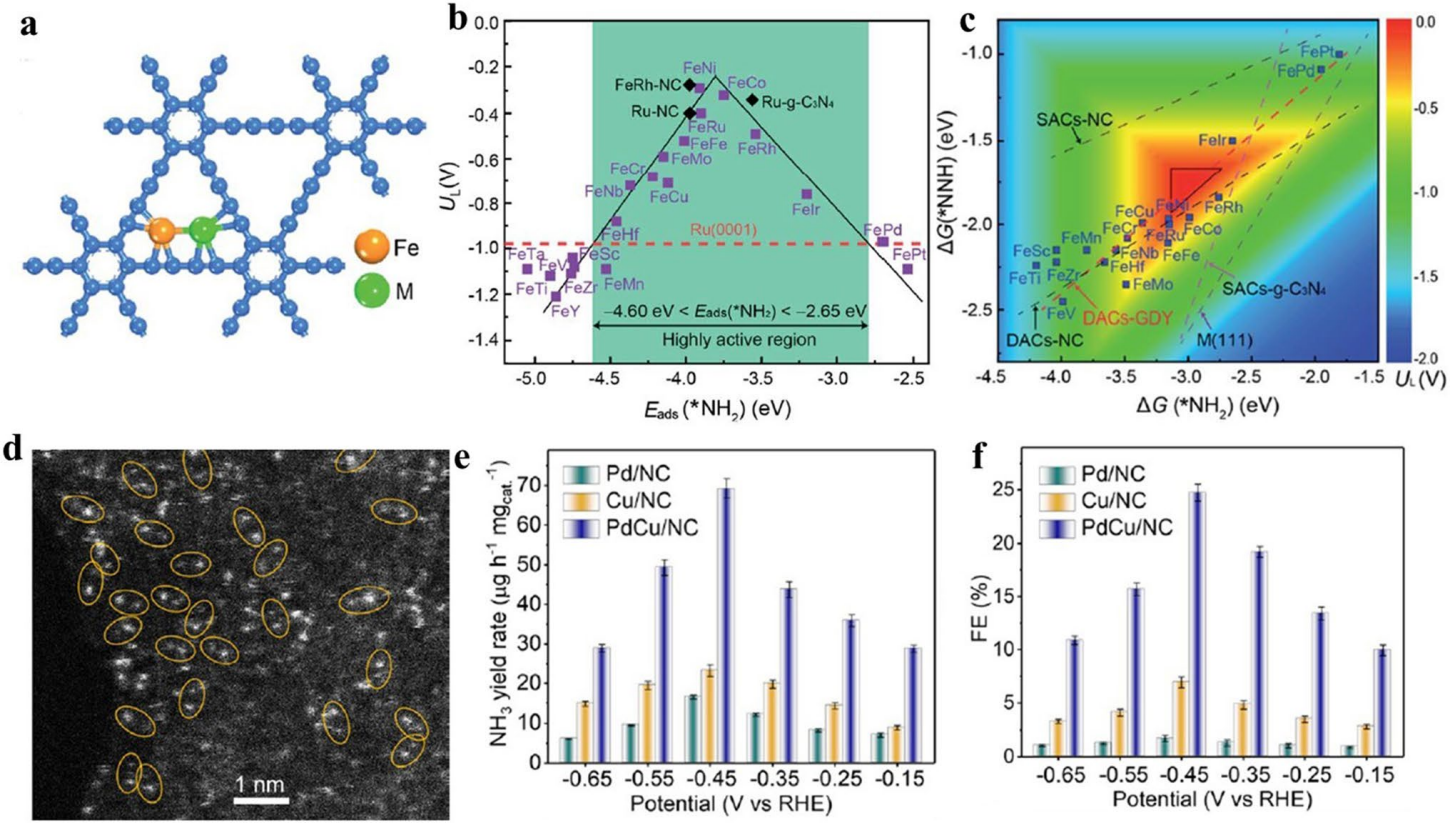
**a** Illustration of FeM-GDYs. **b** Volcano relationship between the *U*_L_ and E_ads_(*NH_2_). **c** Colour contour plot of ΔG(*NNH) and ΔG(*NH_2_) [181]. Copyright 2021 Royal Society of Chemistry. **d** HAADF-STEM image of PdCu/NC. **e** NH_3_ yield rates and **f** FEs of Pd/NC, Cu/NC and PdCu/ NC [184]. Copyright 2021 Wiley–VCH GmbH

The synthesis of NH_3_ by electrochemical NRR includes complex processes involving different mechanisms. The adsorption of nitrogen species on the surface of the electrocatalysts is quite important to the subsequent reduction process. Guo et al. calculated the activity and selectivity of DACs in NRR supported by phthalocyanine to investigate the N_2_ adsorption configurations on different catalyst surfaces [183]. The adsorption energy of N_2_H* was used as the activity descriptor. Three homonuclear and 28 heteronuclear DACs with high activity were selected. In particular, the catalytic activity of Ti_2_-PC, V_2_-Pc, TiV-PC, VCr-Pc and VTa-PC was higher than most reported catalysts under acidic conditions. Xu et al. selected graphdiyne as substrate to high-throughput calculate 26 kinds of Fe-based model DACs (FeM-GDYs) (Fig. 17a) [181]. To identify a more suitable structure, the potential determining step (*U*_L_) and E_ads_(*NH_2_) were calculated and analysed as descriptors. As shown in Fig. 17b, a well-defined volcano-shaped relationship was successfully established between *U*_L_ and E_ads_(*NH_2_). E_ads_(*NH_2_) of approximately − 3.80 eV was found to be the most suitable adsorption energy. Furthermore, a colour contour plot was constructed to investigate the effects of different metal atoms and substrates (Fig. 17c). Among them, FeM-GDYs (M=Ni, Co, Ru) located at the edge of the central region, exhibiting highest activity for NRR. Han et al. designed a Pd–Cu DAC on N-doped carbon (PdCu/NC) for the reason that Cu could accelerate the hydrogen dissociation and the electron transfer rate [184]. As shown in Fig. 17d, HAADF-STEM image clearly revealed the formation of metal dimers. The subsequent NRR performance measurement demonstrated a high NH_3_ yield rate of PdCu/NC reached 69.2 ± 2.5 μgh^−1^ mg cat.^−1^, much higher than Pd/NC and Cu/NC (Fig. 17e). The Faradaic efficiency of PdCu/NC for NRR could reach 24.8 ± 0.8% at − 0.45 V, demonstrating a higher selectivity after introducing Cu atom adjacent Pd site (Fig. 17f).

In addition to metal atoms, substrate effect was also explored to enhance catalytic activity in NRR. He et al. calculated the NRR activity of DACs supported on different substrates [185]. The activity of catalysts supported by different substrate was analysed by the d-band theory. The strong interaction between the Fe_2_ clusters and these two-dimensional substrates leaded to the transfer of the d-band centre. The linear relationship between the E_ads_ of N_2_ and the d-band centre was investigated. In particular, Fe_2_/g-C_3_N_4_ was expected to exhibit superior NRR catalytic activity with a small limiting potential of − 0.32 V. These theoretical studies provide a powerful reference for the design and synthesis of DAC towards NRR in the future.

### Oxygen Evolution Reaction (OER)

Oxygen evolution reaction (OER) as half-reaction of water splitting and rechargeable metal-air batteries, has received much attention in recent years in the field of energy storage and conversion [186–188], which involves multi-step electron transfer steps exhibiting sluggish kinetics [189]. In previous studies, noble metal oxides (RuO_2_ and IrO_2_) have been reported as benchmark OER catalysts, yet suffer from the drawbacks of high cost and low durability [187, 190]. Transition metal SACs are considered as promising alternatives to noble metal oxides showing good catalytic performance in OER [191–193]. The presence of the scaling relations among the intermediates (*OH, *O and *OOH) formed during the OER prevents further reduction of the overpotential [188]. Isolated sites in SACs are limited by scaling relationships that make it difficult to optimize the adsorption of different oxygen-containing intermediates simultaneously. Therefore, DACs with dual sites as well as multiple synergistic interactions are expected to perform better in OER [194, 195].

**Fig. 18:**
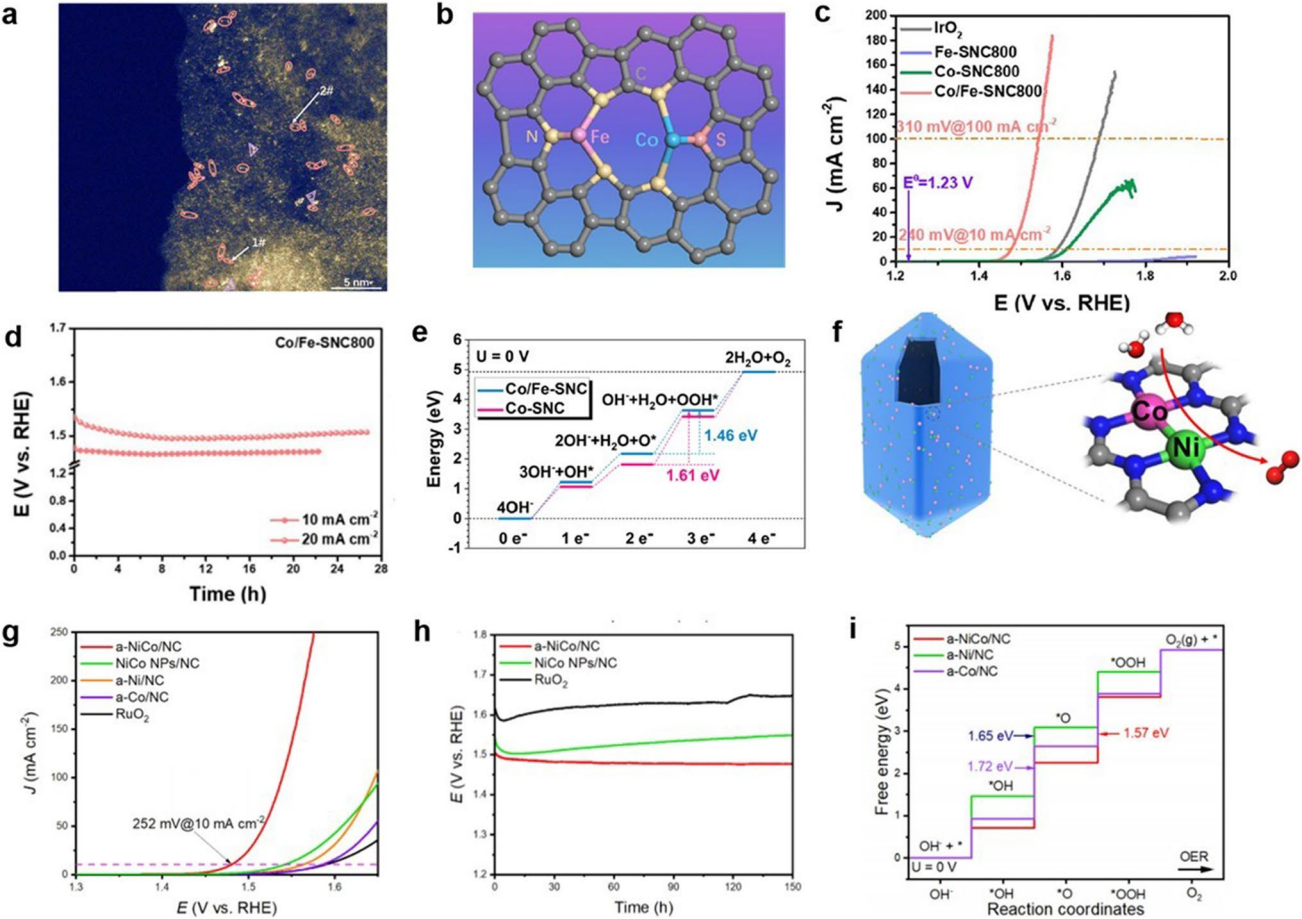
**a** HAADF-STEM images of Co/Fe-SNC800. **b** Illustration of Co/Fe dual-metal site. **c** LSV curves of Fe-SNC800, Co-SNC800, Co/Fe-SNC800 at 1600 rpm. **d** Chronopotentiometry plots of Co/Fe-SNC800. **e** Reaction energy diagram of Co/Fe-SNC and Co-SNC under U = 0 V [196]. Copyright 2023 Royal Society of Chemistry. **f** Illustration of a-NiCo/NC. **g** LSV curves of a-NiCo/NC, NiCo NPs/NC, a-Ni/NC, a-Co/NC, RuO_2_ at 1600 rpm. **h** Chronopotentiometry plots of a-NiCo/NC, NiCo NPs/NC, RuO_2_. **i** Free energy diagram of a-NiCo/NC, a-Ni/NC, a-Co/NC under U = 0 V [85]. Copyright 2022 Wiley–VCH GmbH

To achieve a lower overpotential, Chen et al. prepared a DAC with Co/Fe dual-metal site (denoted as Co/Fe-SNC800) (Fig. 18a) [196]. In the diatomic site, the Fe atom is coordinated with three N atoms, while the Co atom is coordinated with two N atoms and a sulphur atom (Fig. 18b). The Co/Fe-SNC800 exhibited an overpotential of only 240 mV at 10 mA cm^−2^, which was much lower than that of IrO_2_ and the corresponding SACs (Fig. 18c). Co/Fe-SNC800 can work stably at a current density of 20 mA cm^−2^ (Fig. 18d). A strong adsorption between Fe sites and oxygen-containing intermediates was found. At high potential, Fe^2+^ was easily oxidized to form Fe_site_OOH. The Co site was subsequently able to capture *OOH on Fe_site_OOH to form Co_site_OOH, which greatly reduced the formation energy barrier of Co_site_OOH. Further theoretical simulations demonstrated the formation of *OOH as RDS. The synergetic interaction between the Co site and Fe site in the Co/Fe-SNC800 reduced the formation energy barrier of OOH* on the single-atom Co site from 1.61 to 1.46 eV, which significantly accelerated the OER process (Fig. 18e). Pei et al. synthesized a DAC (a-NiCo/NC) with a hollow prismatic morphology with Ni-Co diatomic sites (Fig. 18f) [85]. The prepared a-NiCo/NC exhibited an overpotential as low as 252 mV at a current density of 10 mA cm^−2^, which was significantly lower than that of the prepared single-atom Ni and single-atom Co catalysts (Fig. 18g). a-NiCo/NC was able to operate continuously in alkaline solution for 150 h without an increase in overpotential in durability tests (Fig. 18h). DFT calculations show a strong electronic coupling between Ni and Co atoms in a-NiCo/NC. Compared with the single-atom Co and single-atom Ni sites, the d-band centres of both Co and Ni in a-NiCo/NC have risen to the Fermi energy level, which is favourable for the adsorption of the active sites to the OER reaction intermediates. The free energy diagram reveals that the interaction of Ni and Co leads to a shift in the rate-determining step from O* formation to OOH* formation and a significant decrease in the reaction overpotential, resulting in a better OER performance (Fig. 18i).

### Other Reactions

Comparing DACs with SACs based on nitrogen-doped carbon substrate, we can see that the DACs could have higher metal contents and sometimes have metal–metal bonds, which imply that they should be more stable under relatively reductive conditions, including ORR, CO_2_RR, NRR, and HER. However, this does not mean DACs cannot be used for oxidative reactions. Alkaline exchange membrane fuel cells (AEMFCs) have shown faster electrochemical kinetics, lower catalyst cost, and weaker corrosion than proton exchange membrane fuel cells (PEMFCs), providing more possibilities for the use of nonprecious metal catalysts [197, 198]. However, hydrogen oxidation reaction (HOR) kinetics in alkaline electrolytes are 2–3 orders of magnitude slower than in acidic electrolytes [199, 200]. DACs with tunable electronic structure can optimize the surface binding energy of relevant adsorbates in HOR and thus are considered as a possible facilitator of HOR kinetics. In order to find promising DACs for HOR, Han et al. selected metal atoms (Ru, Ni, Pd and Ir) with suitable *H binding energy for the composition of different diatomic pairs and performed theoretical calculations [201]. As key adsorbents affecting HOR in alkaline electrolytes, the binding energies of both *H and OH* were calculated in detail. Ru–Ni was identified as the best combination owing to the simultaneous *H binding energy and *OH binding energy closest to 0 eV. Therefore, DACs with Ru–Ni dual-metal sites anchored on N-doped carbon (denoted as RuNi/NC) were prepared and characterized. In agreement with the results of theoretical calculations, the catalytic activity of RuNi/NC in 0.1 M KOH electrolyte was superior than that of all samples including benchmarked Pt/C.

**Fig. 19:**
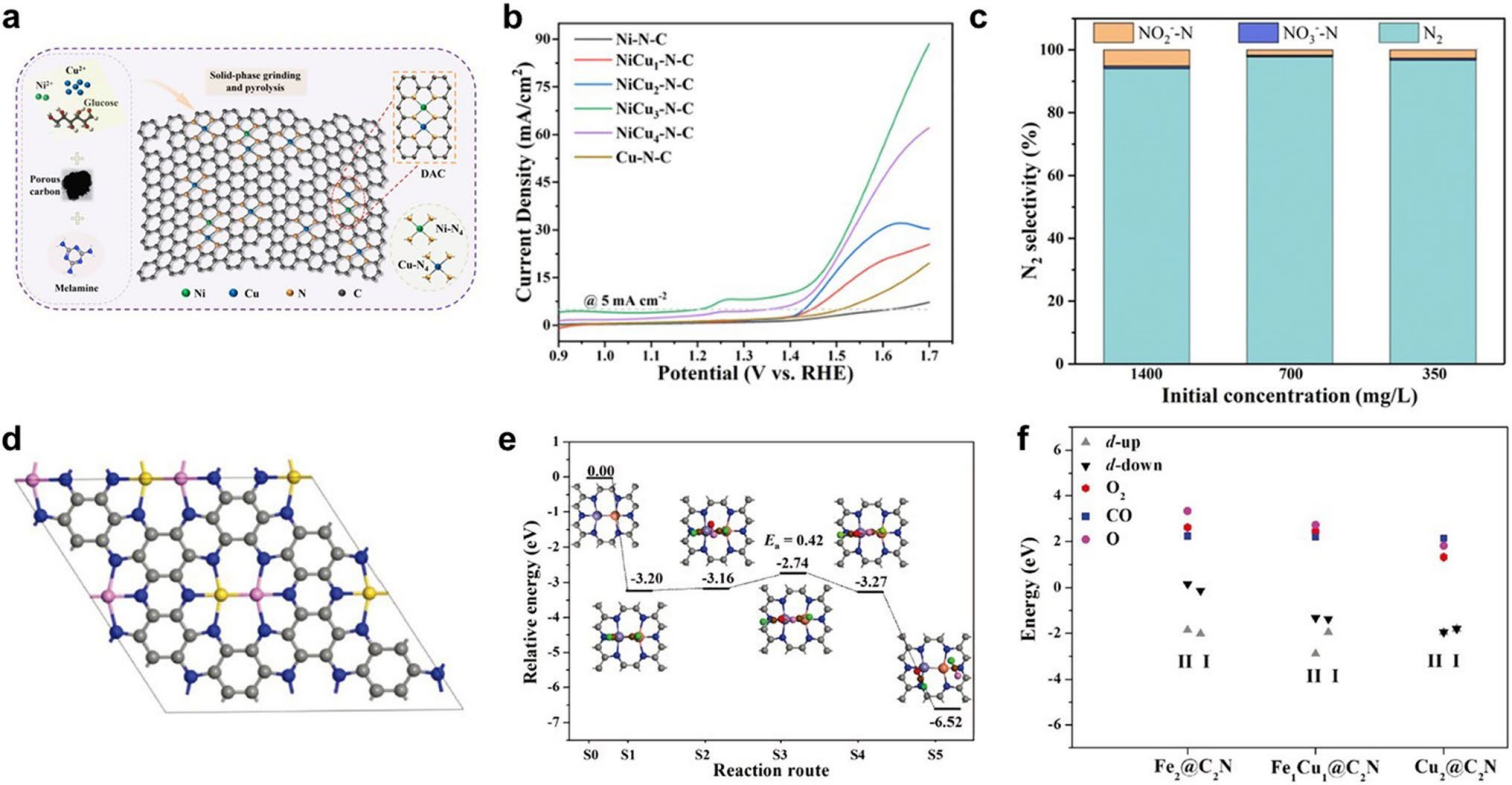
**a** Schematic synthesis of NiCu_3_–N–C DAC**. b** LSV curves in 1 M NaOH + 0.2 M NH_4_Cl.** c** N_2_ selectivity of NiCu_3_–N–C DAC in different initial concentrations of ammonia. Copyright 2023 Elsevier Ltd.** d** Preferable configuration of two metal atoms anchored C_2_N (C, grey; N, blue; gold, M^1^; pink, M^2^). **e** Reaction pathways based on the TER mechanism. **f** *d*-band centres and the adsorption energies of CO, O_2_, and O for the Fe_2_@C_2_N, Fe_1_Cu_1_@C_2_N and Cu_2_@C_2_N [209]. Copyright 2019 Wiley–VCH GmbH

Excessive discharge of nitrogen-containing pollutants has led to many environmental problems. Electrochemical ammonia oxidation reaction (AOR) is considered an ideal solution for the treatment of ammonia in wastewater because of its economic and environmental advantages [202, 203]. However, the stabilized N–H bond (99.5 kcal mol^−1^) in ammonia leads to sluggish reaction kinetics [204, 205]. In addition, the products of AOR may not only be N_2_, but may also contain NO_*X*_ as additional pollutants due to excessive oxidation [206, 207]. To solve the above problem, Zhang et al. prepared a DAC (NiCu_3_–N–C DAC) with Ni–N_4_/Cu–N_4_ sites (Fig. 19a) [208]. Adjacent metal sites facilitate assisted N–N bond formation for highly selective oxidation of NH_4_ to N_2_. As shown in Fig. 19b, the NiCu_3_–N–C DAC is the most active in AOR, featuring a more negative onset potential and the ability to achieve larger current density. In addition, high selectivity for N_2_ products was demonstrated in different concentrations of ammonia-containing solutions (Fig. 19c), validating the potential application of DACs in AOR.

Oxidation of CO in polymer electrolyte fuel cells plays an important role in reducing environmental CO emissions and removing CO pollution from H_2_ fuel gases. The conversion and elimination of CO has been widely studied. One of the effective ways is to oxidize CO to CO_2_. SACs have shown considerable potential for CO oxidation [210]. As an extension of SACs, DACs showed optimized interactions with the intermediates during CO oxidation, leading to widespread concern. Li et al. evaluated the potential of heteronuclear metal dimer towards CO oxidation by DFT calculations (Fig. 19d, e) [209]. The CO/O_2_ adsorption and CO oxidation pathways of heteronuclear DAC Fe_1_Cu_1_@C_2_N was systematically studied. The TER mechanism with two CO molecules preabsorbed on the surface was the most favourable because it demonstrated the lowest activation energy. According to the TER mechanism, the reaction begins with two adsorbed CO (OC*) and a close O_2_. O_2_ is activated by two adsorbed CO, and in the transition state S3, the O–O bond is elongated to 1.34 Å. After the transition state S3, a pentagonal ring intermediate OCOOCO was formed, in which the O–O bond extended further to 1.46 Å. The two newly formed C–O bonds were 1.36 and 1.38, respectively. The intermediate product then spontaneously decomposes into two CO_2_ molecules, simultaneously releasing an energy of 3.25 eV. In addition, Fe_1_Cu_1_@C_2_N showed an optimized d-band centre and adsorption strength of CO/O_2_/O compared with Fe_2_@C_2_N and Cu_2_@C_2_N (Fig. 19f). The theoretical results suggested that constructing dual-metal sites could efficiently regulate d-band centre, thus improving the activity of SACs towards CO oxidation.

## Summary and Prospect

To summarize, metal atoms in the atomic catalyst demonstrate high unsaturated coordination, quantum confinement, and strong metal interaction, resulting in extremely remarkable catalytic performance. In particular, DACs, as one of the hottest atomically dispersed catalysts nowadays, show great potential for energy-related small molecule electrocatalytic reactions due to their flexible and tunable geometrical structures and electron configurations. Synergistic interactions between metal sites in DACs provide more opportunities for the enhancement of catalytic activity of DACs as well as application promotion. DACs are currently being extensively studied and have shown great potential in various energy-related fields. Although some breakthroughs in DAC research have been achieved, they are still at the initial stage. In order to achieve the promotion and practical application of DACs, more efforts must be devoted to the following challenges.

1. DACs are in urgent need of more precise synthesis approaches. Low-cost, universal, and efficient strategies for the synthesis of DACs need to be further developed in order to meet the needs of industrial production. Although different strategies have been developed for the synthesis of DACs, it is still difficult to meet the experimental expectations due to the precise structure of DACs. Most current syntheses of DACs rely on a high-temperature pyrolysis step, which makes it difficult to precisely modulate the active site configuration. In addition to the desired diatomic sites, it is difficult to avoid the formation of many unintended single-atomic sites in the as-prepared catalysts. Besides, unexpected homonuclear diatomic sites may also arise during the preparation of heteronuclear DACs. Although technique like ALD has been developed for the precise synthesis of DACs at atomic level, they have been difficult to scale up due to the cost of equipment and the complexity of the process. Therefore, more research is needed to develop low-cost, versatile, and efficient strategies to precisely synthesize DACS.
2. To accurately characterize DACs remain a challenge. For the fine structural characteristics of DAC, there are many challenges in characterization. Conventional electron microscopy techniques such as SEM and TEM are unable to identify DACs from the atomic scale. Advanced electron microscopy techniques such as AC-HAADF-STEM face the limitation of high cost. Besides, the AC-HAAD-STEM technique relies on high Z-contrast to identify diatomic sites, but cannot distinguish elements that are not sufficiently different in atomic number. Similarly, XAS is considered a powerful tool for characterizing the electronic structure and geometric configuration of DACs. However, XAS reveals the average information of the test element in the sample and thus may not be accurate in judging local structure. In general, it is difficult to accurately analyse the structural configuration of DACs at the atomic level with a single characterization. Additional characterization methods should be developed and combined to provide a comprehensive and in-depth understanding of DACs.
  On the other hand, DACs need to be characterized in a more efficient way to meet the practical application. In addition to the scarce AC-STEM and XAS techniques, it is practicable to develop new determinations to describe the formation of DACs. For example, Fourier transform infrared spectroscopy has shown great potential for characterizing DACs. It is able to discover the unique bridged-bonded configuration of the adsorbate on DACs, thus demonstrating the formation of diatomic sites.
3. It is difficult to study the mechanism of different DACs for various reactions. The study of the reaction mechanism is mainly carried out by theoretical simulations combined with experimental methods. On the theoretical side, it is important to determine what role the different DACs play in a particular reaction process, such as altering the reaction path, modulating selectivity, etc. Although these have been explored in the current work, it is still not enough to establish a systematic understanding of the structure–activity relationships, and thus theoretical calculations can yet provide only a limited reference to guide the design of DACs. In addition, in-depth studies of the complex reaction conditions under actual operating conditions are still absent. Furthermore, screening the most suitable configuration of DACs based on the structure–activity relationships also presents significant challenges. In addition to the abundant and diverse metal combinations of DACs, the corresponding coordination environment and metal–support interactions are also important parameters affecting the catalytic activity. DACs are difficult to design to optimize such complex parameters simultaneously. Although theoretical simulations and machine learning can screen the DACs that meet the requirements, the model built is idealized and thus does not fully satisfy the actual experimental situation. Therefore, to design more efficient DACs, it is necessary to create more samples for reference and to discover suitable descriptors to coordinate the above-mentioned parameters that determine activity.
  On the experimental side, in situ characterization techniques under actual operating conditions are desired to obtain more reliable reaction mechanisms and can be verified with the theoretical calculations. The current in situ characterization techniques have made some breakthroughs in the study of reaction mechanisms; however, the mechanism studies are always limited to laboratory electrochemical tests and difficult to consider the actual operating environment of the relevant energy devices. More theoretical simulations and in situ characterization techniques for operational conditions should be further developed to establish more applicative mechanistic perception.
4. The durability of DACs in different applications still needs to be improved. We note that the current strategies for regulating the durability of DACs focus on the following aspects: (i) screening of metal atom species in DACs. Li et al. performed theoretical calculations for 335 DACs and predicted the structure–stability relationships of DACs in terms of resistance to forming single-atom sites, metal atom aggregation into nanoparticles and electrochemical dissolution [211]. For example, in HER, all calculated DACs have good durability, whereas in OER only very few species of metal atoms can form stable DACs. (ii) Regulating the coordination environment of metal atoms. Wang et al. realized the enhancement of oxidation resistance and corrosion resistance by introducing P atoms into the coordination environment of Fe-Co DAC [80]. (iii) Substrate stabilization. Zhao et al. demonstrated through theoretical simulations that the hybridization between the 2*P* orbitals of N and the 3*d* orbitals of the transition metals present in the C_2_N substrate can inhibit the diffusion of the metal atoms and thus achieve high stability [212]. Wang et al. prepared axial DACs with vertically stacked graphene as the substrate, and they successfully improved the structural stability of the DACs with the help of the three-dimensional confinement effect of the substrate [213].
  The durability of DACs under actual operating conditions has not been studied enough. Most of the current works on DACs have focused on catalytic activity and selectivity, while there is a lack of sufficient knowledge on the durability. On the one hand, the durability measurement of DACs always remains in the laboratory electrochemical testing stage, whereas the real durability should take into account the actual working conditions in the related energy devices. On the other hand, the lack of in-depth exploration of the deactivation, poisons, and degradation mechanisms of DACs is not favourable to the development of targeted optimization of the durability. In order to realize the practical application of DACs, durability research is essential, which should be one of the key research directions for DACs in the future.
5. New measurement systems need to be developed to more effectively assess the catalytic performance of DACs. In addition to a more accurate identification of the intrinsic activity, more assessment indicators need to be discovered for closing the knowledge gap between the activity of DACs in the laboratory and their performance in practical applications. It is also significant to construct the devices with reference to real applications and to evaluate the catalysts under operating conditions according to industrial requirements.

In conclusion, although there are still many research gaps in DACs, their merits and great potential will undoubtedly make them an important branch in the field of catalysis in the future.
